# Deletion of *Cryptococcus neoformans* AIF Ortholog Promotes Chromosome Aneuploidy and Fluconazole-Resistance in a Metacaspase-Independent Manner

**DOI:** 10.1371/journal.ppat.1002364

**Published:** 2011-11-17

**Authors:** Camile P. Semighini, Anna F. Averette, John R. Perfect, Joseph Heitman

**Affiliations:** 1 Department of Molecular Genetics and Microbiology, Duke University Medical Center, Durham, North Carolina, United States of America; 2 Department of Medicine, Duke University Medical Center, Durham, North Carolina, United States of America; 3 Department of Pharmacology and Cancer Biology, Duke University Medical Center, Durham, North Carolina, United States of America; University of Melbourne, Australia

## Abstract

Apoptosis is a form of programmed cell death critical for development and homeostasis in multicellular organisms. Apoptosis-like cell death (ALCD) has been described in several fungi, including the opportunistic human pathogen *Cryptococcus neoformans*. In addition, capsular polysaccharides of *C. neoformans* are known to induce apoptosis in host immune cells, thereby contributing to its virulence. Our goals were to characterize the apoptotic signaling cascade in *C. neoformans* as well as its unique features compared to the host machinery to exploit the endogenous fungal apoptotic pathways as a novel antifungal strategy in the future. The dissection of apoptotic pathways revealed that apoptosis-inducing factor (Aif1) and metacaspases (Mca1 and Mca2) are independently required for ALCD in *C. neoformans*. We show that the apoptotic pathways are required for cell fusion and sporulation during mating, indicating that apoptosis may occur during sexual development. Previous studies showed that antifungal drugs induce ALCD in fungi and that *C. neoformans* adapts to high concentrations of the antifungal fluconazole (FLC) by acquisition of aneuploidy, especially duplication of chromosome 1 (Chr1). Disruption of *aif1*, but not the metacaspases, stimulates the emergence of aneuploid subpopulations with Chr1 disomy that are resistant to fluconazole (FLC^R^) in vitro and in vivo. FLC^R^ isolates in the *aif1* background are stable in the absence of the drug, while those in the wild-type background readily revert to FLC sensitivity. We propose that apoptosis orchestrated by Aif1 might eliminate aneuploid cells from the population and defects in this pathway contribute to the selection of aneuploid FLC^R^ subpopulations during treatment. Aneuploid clinical isolates with disomies for chromosomes other than Chr1 exhibit reduced *AIF1* expression, suggesting that inactivation of Aif1 might be a novel aneuploidy-tolerating mechanism in fungi that facilitates the selection of antifungal drug resistance.

## Author Summary

Fungal pathogens can cause life-threatening diseases, and the infections that they cause are notoriously difficult to treat. Despite the availability of antifungal drugs, most inhibit fungal growth but do not consistently or efficiently eliminate the pathogen. In addition, fungal cells are very similar to human cells, and therefore, many of the available antifungal agents have toxic side effects. Thus, more efficient drugs with less adverse effects are clearly needed. We investigated apoptosis, a process in which cells become programmed to commit suicide, in the pathogenic fungus *Cryptococcus neoformans*. We studied genes that regulate apoptosis in *C. neoformans* and, after inactivating three genes involved in this pathway, we observed defects in sexual reproduction. Such mating defects decrease the production of spores, which are inhaled and cause cryptococcal disease. We also showed that the absence of one investigated apoptotic gene, *aif1*, resulted in the selection of antifungal-resistant pathogens (when the fungal cells no longer respond to the drug), which makes treatment of the disease more difficult. The discovery of drugs that kill fungal cells specifically without affecting the cells of the patient being treated holds great potential. Therefore, triggering apoptosis should be further investigated as a new approach to treat fungal pathogens.

## Introduction

Apoptosis is a form of programmed cell death critical for development and homeostasis in multicellular organisms [1]. The mechanisms of apoptosis are complex and energy-dependent. Numerous pro-and anti-apoptotic signals have to be integrated to activate apoptotic effectors only when necessary. Mitochondria play an important role in apoptosis by releasing several proteins, such as cytochrome c, which triggers the proteolytic maturation of caspases [2]. Caspases are a family of cysteine proteases that are recognized as key components of the apoptotic machinery. Caspases proteolytically cleave specific substrates, leading to the ordered dismantling of intracellular components during apoptotic death [3]. Caspase-independent apoptotic pathways involve the translocation of two nucleases, apoptosis-inducing factor (AIF) and endonuclease G (EndoG), from the mitochondria to the nucleus [4].

Features typical of apoptotic cell death, including chromatin fragmentation and condensation and translocation of phosphatidylserine to the outer layer of the cellular membrane, were described for the first time in fungi in a *Saccharomyces cerevisiae cdc48* mutant [5]. Heterologous expression of mammalian pro- or anti-apoptotic Bax or Bcl2 in yeast causes or prevents apoptosis-like cell death (ALCD), respectively [6]–[8]. Subsequently, several fungal species have been reported to undergo ALCD as a consequence of interactions with other organisms and during development and aging (reviewed by [9]–[13]). The identification and functional analysis of core components of the apoptotic machinery has revealed partial conservation, along with substantial differences in function and mode of action between fungal and human proteins. Consequently, apoptotic pathways are considered suitable targets for novel antifungal therapies.

*Cryptococcus neoformans* var. *neoformans* undergoes ALCD when co-cultivated with the bacterium *Staphylococcus aureus* and also in response to oxidative stress induced by hydrogen peroxide [14]. Conversely, purified capsular polysaccharides from *C*. *neoformans* are known to induce apoptosis in host immune cells, including rat splenocytes [15], [16], activated human T cells [17]–[19], and murine macrophages [20]–[22]. Apoptosis induction is a strategy that *C. neoformans* may deploy to evade host innate immune responses and contributes to the powerful immunosuppression that accompanies cryptococcosis. Because *C. neoformans* both induces and undergoes apoptosis in response to different signals, its apoptotic machinery must have unique features compared to the apoptotic-signaling cascade of the host, which are potential new therapeutic targets.

*C. neoformans* has emerged as an important pathogen of immunocompromised and immunocompetent patients, estimated to cause 1 million new cryptococcal disease cases globally each year, with up to 600,000 fatalities yearly [23]. Cryptococcosis is a systemic mycosis that commonly involves the lungs and central nervous system. Untreated cryptococcal meningitis has a mortality rate of 100%. The recommended therapy for cryptococcal meningitis, which is intravenous amphotericin B (AMB) combined with flucytosine [24], is associated with high toxicity and serious adverse reactions in patients. Moreover, the high costs associated with AMB therapies limit its availability in resource-limited regions such as sub-Saharan Africa. Instead, a less effective agent, fluconazole (FLC), is widely used in these areas, and mortality remains high. FLC is a triazole antifungal drug that inhibits the fungal cytochrome P450 lanosterol 14α-demethylase (encoded by *ERG11*), thereby blocking the production of ergosterol, the main sterol of fungal membranes.

Here we studied the apoptotic pathways of *C. neoformans* aiming to identify unique features to specifically induce ALCD of this pathogen as a novel treatment. We found an unexpected connection between ALCD and antifungal drug resistance: the elimination of the apoptosis-inducing factor (Aif1) stabilizes aneuploidy and promotes the generation of FLC-resistance. Selection of FLC^R^ populations with Aif1 defects occurs in vivo, and the inactivation of Aif1-mediated ALCD in *C. neoformans* may explain cases of FLC treatment failures in the clinical setting. In addition, apoptotic pathways are involved in sexual development and the formation of spores, which serve as infectious propagules of *C. neoformans*.

## Results

### Characterization of apoptotic pathways in *Cryptococcus neoformans*

**Figure 1:**
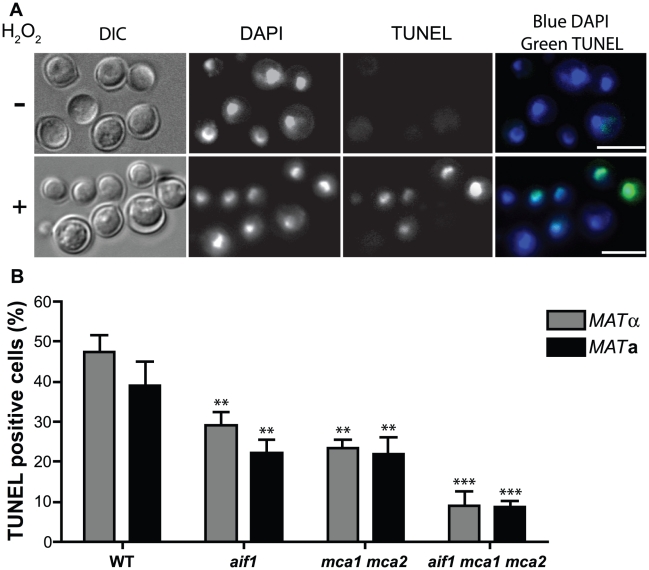
Apoptotic pathways are conserved in *C. neoformans*. A . TUNEL assay indicating apoptosis-like cell death occurs following exposure to hydrogen peroxide. Cells of wild-type serotype A H99 strain grown on YNB media treated for 3 hr with 0 (upper panels) or 2 mM (lower panels) H_2_O_2_ were labeled with DAPI (blue fluorescence) and TUNEL (green fluorescence) and analyzed by fluorescence microscopy. Bars, 10 µm. **B**. Percentage of TUNEL positive cells in wild-type H99 control and *aif1*, *mca1 mca2*, and *aif1 mca1 mca2* mutant strains treated with 2 mM H_2_O_2_ and analyzed by flow cytometry. Data represent mean ± standard deviation. ** and *** indicates significance at p<0.01 and 0.001 compared to H99 strain (WT *MAT*α), respectively.

ALCD has been described in several fungal species and is induced by different compounds and stress conditions (reviewed by [9], [11]–[13]). In particular, hydrogen peroxide has been reported to trigger ALCD in *C. neoformans* var. *neoformans* [14], and so we tested if similar conditions would induce cell death in *C. neoformans* var. *grubii*, which is responsible for more than 90% of cryptococcal infections worldwide [25]. Treatment of the H99 sequence reference strain with 2 mM hydrogen peroxide for 3 hours at 37^o^C induced characteristic apoptotic markers, such as DNA fragmentation indicated by TUNEL (TdT-mediated dUTP nick end labeling). TUNEL detects DNA fragmentation by using terminal deoxynucleotidyl transferase (TdT) to incorporate dUTP tagged with FITC into the blunt ends of double-stranded DNA breaks, and is the standard method for identification and quantification of apoptotic cells [26]. While TUNEL-FITC positive cells were only rarely observed in untreated controls (Figure 1A, upper panels), ∼45% of wild-type cells showed DNA degradation after treatment with hydrogen peroxide (Figure 1B).

We also used Annexin V-FITC to bind to phosphatidylserine (PS), a plasma membrane phospholipid that is externalized to the outer layer during apoptosis. Annexin V-FITC does not bind cells with an intact plasma membrane, but can falsely detect the PS present in the inner membrane of lysed (necrotic) cells. Therefore, simultaneous staining with the vital dye propidium iodide (PI) allows the discrimination of early apoptotic (FITC+, PI−) and late apoptotic or necrotic yeast cells (FITC+, PI+). The percentage of WT cells staining positive with Annexin V-FITC but negative with PI (apoptotic cells) rose to ∼42% after treatment with hydrogen peroxide (Figure S1A, green bars).

To find potential regulators of ALCD in *C. neoformans*, functional homologs of the major apoptotic machinery identified from *S. cerevisiae* were used to search the H99 genome database. The following homologs were identified as first reciprocal BLASTp hits: cytochrome c oxidase subunit 1 (*COX1*, CNAG_09009); inhibitor of apoptosis protein (*IAP1*, CNAG_04708), and the caspase-independent nucleases apoptosis-inducing factor (*AIF1*, CNAG_04521) and endonuclease G (*ENDOG* CNAG_02204). Two metacaspases were identified in the H99 genome using the *S. cerevisiae* Mca1p: *MCA1* (CNAG_04636; 60% identity and 4e-103) and *MCA2* (CNAG_06787, 44% identity and 1e-88). Interestingly, no apparent high-temperature resistance A homolog (HtrA2, also called Nma111p in yeast for nuclear mediator of apoptosis) was found. *C. neoformans* does not have apparent homologs of the apoptotic protease activating factor (APAF) and the poly(ADP-ribose) polymerase (PARP), which are present in filamentous fungi but absent in *S. cerevisiae*. Of note, multiple attempts to disrupt the *COX1* gene were unsuccessful (Toffaletti and Perfect, unpublished observations) and it may therefore be an essential gene in *C. neoformans*.

We generated two independent *aif1*, *mca1 mca2*, and *aif1 mca1 mca2* null mutants in congenic α and **a** *C. neoformans* var. *grubii* backgrounds to address whether *C. neoformans* has parallel caspase-dependent and caspase-independent apoptotic pathways. After treatment with 2 mM hydrogen peroxide, the *aif1* mutants showed a 41% reduction, and *mca1 mca2* showed a 47% reduction in TUNEL-positive cells when compared to wild-type (Figure 1B), while the *aif1 mca1 mca2* triple mutant showed a reduction of 79%. In addition, all mutant strains showed reduced externalization of PS revealed by Annexin V assay (Figure S1A) and did not show increased vacuolization observed during autophagic cell death (not shown). Finally, the *aif1*, *mca1 mca2*, and *aif1 mca1 mca2* null mutants were more resistant to 2 mM hydrogen peroxide than the wild-type (Figure S2). Our results indicate that Aif1 and the two metacaspases independently promote ALCD in *C. neoformans*.

### Aif1 inactivation potentiates fluconazole-resistance and aneuploidy

Amphotericin B (AMB) and fluconazole (FLC) are antifungal drugs widely used to treat major fungal diseases. Recent treatment guidelines recommend the more toxic AMB-based regimens for induction therapy instead of the better-tolerated FLC-based regimens to treat cryptococcal meningitis, because the latter is fungistatic and because *Cryptococcus* strains repeatedly exposed to FLC can acquire direct drug resistance [27]. AMB was shown to induce ALCD in *Aspergillus fumigatus* [28] and *Candida albicans* in vitro [29], and in biofilms of *C. albicans*, *Candida krusei*, and *Candida parapsilosis* [30]. To test if antifungal drugs would induce ALCD in *C. neoformans*, we first determined if the apoptotic mutants have increased resistance to AMB and FLC. Using Epsilometer test strips (Etest), the *aif1* and *mca1 mca2* mutants showed no significant difference in sensitivity to AMB (Table 1) and we could not detect ALCD induction by treating wild-type cells with the minimal inhibitory concentration of 0.125 µg/ml of AMB (Figure S1B). On the other hand, the *aif1* and *mca1 mca2* mutants showed increased resistance to FLC (Table 1). Even though FLC is a fungistatic agent, we tested if this azole was able to induce ALCD in *C. neoformans*. We could not detect ALCD induction by treating wild-type cells with up to 64 µg/ml of FLC (Figure S1B and data not shown) and therefore, the increased resistance of the *aif1* and *mca1 mca2* mutants is likely not attributable simply to a lack of cell death induction.

**Table 1:** Minimal inhibitory concentration (MIC, µg/ml) found in Etest assays.

Strain	FLC (µg/ml)	AMB (µg/ml)
H99	6	0.125
CPS3 (*aif1::NAT*)	16*	0.125
CPS70 (*aif1::HYG*)	16*	0.125
CPS174 (*aif1::NAT AIF1-NEO*)	6	0.125
YPH104 (*mca1 mca2*)	24	0.19
CPS89 (*aif1 mca1 mca2*)	8*	0.19
HC-4**	4	0.75
HC-6**	16	0.19
HC-9**	12	0.25
RCT 50**	32	0.125
RCT 52**	>256	0.125
RCT 55**	48	0.19
RCT 17**	16*	0.125
CPS175 (RCT 17 *AIF1-NEO*)	16	0.125

*Rapid acquisition of resistance to FLC.

**Initial cultures of clinical isolates used for Etest assays were obtained directly from frozen CSF samples of patients that had not yet been treated with any antifungal drug (HC isolates from Duke patients; RCT isolates from patients in South Africa).

**Figure 2:**
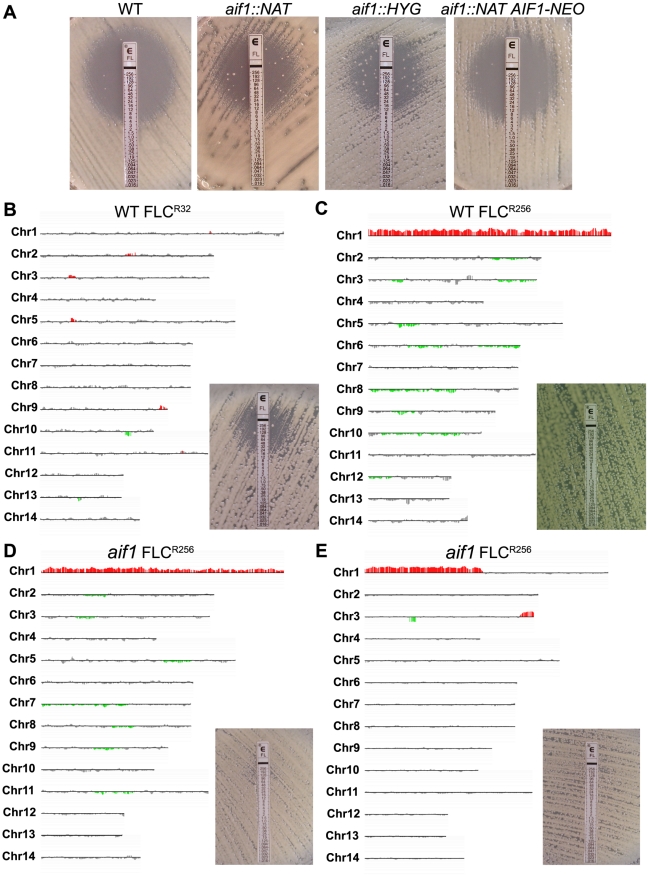
Disruption of apoptosis-inducing factor stimulates the selection of fluconazole resistant subpopulations. A . Etest strip assays showing that deletion of *aif1* increases the frequency of the intrinsic adaptive fluconazole heteroresistant phenotype of serotype A wild-type strain H99. **B**. aCGH analysis of a fluconazole-resistant colony isolated from the H99 wild-type background (CPS51) on an Etest assay showing a higher MIC (32 µg/ml) but no alteration in chromosome numbers. **C**. aCGH analysis of a FLC^R^ clone CPS106, isolated from the CPS51 background on Etest assay showing complete resistance to FLC (MIC >256 µg/ml) and Chr1 disomy. **D-E**. Clones isolated from the *aif1* mutant immediately show complete resistance to FLC (MIC >256 µg/ml) and multiple whole or partial chromosome duplications. Clone CPS18 is disomic for Chr1 (**D**) and clone CPS24 has segmental duplications of chromosomes 1 and 3 (**E**). Vertical bars in red indicate amplifications, and in green deletions, that are statistically significant. Vertical bars in gray indicate no DNA copy number alteration or amplifications or deletions that did not meet the statistical settings.

We also noticed that considerably more FLC^R^ colonies grew within the zone of inhibition around the FLC strip in Etest assays of two independent *aif1* deletion mutants, *aif1::NAT* and *aif1::HYG* (Figure 2A), a phenomenon called heteroresistance. Heteroresistance to azoles, or the presence of drug-resistant cells within a drug-sensitive population, was previously observed as an intrinsic resistance mechanism in all serotypes of *C. neoformans* [31]–[33] and *C. gattii* [34]. During these Etest experiments, resistant colonies within the zone of inhibition were observed up to 10 times more frequently in the *aif1* deletion mutants (Figure 2A). Fluctuation analysis using 12 independent colonies estimated that the resistance rate to 32 µg/ml FLC is about 5 times higher in the *aif1* mutant compared to H99 (Table 2). This is not due to a higher mutation rate in the *aif1* strain because it showed similar 5-FOA^R^ rates compared to the wild-type stain (5.1 and 4.5×10^−8^ respectively).

**Table 2:** FLC resistance rates.

Strain	FLC^R^ rate x 10^-6^ (95% CI*)	FLC^R^ rate relative to WT
WT	4.3 (3.0–6.6)	1
*aif1*	22.2 (17.4–27.5)	5.2
*aif1*+*AIF1*	4.5 (2.8–6.4)	1.1
RCT 17	82.1 (62.7–99.8)	19.3
RCT 17+*AIF1*	14.7 (11.0–17.2)	3.4

*Confidence interval.

H99 was recently reported to be able to adapt to FLC concentrations higher than its minimal inhibitory concentration (MIC) by becoming aneuploid [35]. Chromosome 1 (Chr1) disomy was a common signature of FLC^R^ strains, and two of the genes resident on this chromosome were shown to be important for FLC resistance: *ERG11* (cytochrome P450 lanosterol 14α-demethylase), which is the target of FLC, and *AFR1* (antifungal resistance 1, [36]), which is the major ATP binding cassette (ABC) transporter of azoles in *C. neoformans* [35]. Therefore, we determined the copy number of Chr1 in several FLC^R^ colonies isolated from independent Etest assays (Table 3). We used a quantitative polymerase chain reaction (qPCR) assay and three probes distributed along Chr1: *ERG11*, *ACT1*, and *AFR1* (Figure S3). A subset of the colonies was karyotyped by array comparative genomic hybridization (aCGH; Figure 2B-E and Figure S4). DNA content analyses by flow cytometry indicate that all strains described in Table 3 are haploid or near haploid (n+1) (data not shown).

**Table 3:** Summary of ploidy of FLC^R^ clones.

Background*	Strain	FLC MIC (µg/ml)	Genotype**	Method
H99 FLC^R6^	CPS51	32	n	aCGH
H99 FLC^R6^	CPS52	16	n	aCGH
H99 FLC^R6^	CPS53	64	n+(1)	qPCR
H99 FLC^R6^	CPS105	32	n	qPCR
H99 FLC^R6^	CPS137	24	n	qPCR
H99 FLC^R6^	CPS151	48	n+(1)	qPCR
H99 FLC^R6^	CPS154	48	n+(1)	aCGH
H99 FLC^R6^	CPS155	64	n+(1)	qPCR
CPS51	CPS106	>256	n+(1)	aCGH
CPS51	CPS107	>256	n+(1)	qPCR
CPS52	CPS108	192	n+(1)	qPCR
CPS52	CPS109	192	n+(1)	qPCR
CPS105	CPS147	>256	n+(1)	qPCR
CPS108	CPS148	>256	n+(1)	qPCR
CPS109	CPS149	>256	n+(1)	aCGH
*aif1::NAT* FLC^R16^	CPS18	>256	n+(1)	aCGH
*aif1::NAT* FLC^R16^	CPS21	>256	n+(1)	qPCR
*aif1::NAT* FLC^R16^	CPS24	>256	n+dup(1)(3)	aCGH
*aif1::NAT* FLC^R16^	CPS26	>256	n+(1)	qPCR
*aif1::NAT* FLC^R16^	CPS28	>256	n+(1)	qPCR
*aif1::NAT* FLC^R16^	CPS35	192	n+(1)	qPCR
*aif1::NAT* FLC^R16^	CPS37	>256	n+(1)	aCGH
*aif1::NAT* FLC^R16^	CPS48	>256	n+(1)(4)	aCGH
*aif1::NAT* FLC^R16^	CPS49	>256	n+(1)	qPCR
*aif1::HYG* FLC^R16^	CPS110	>256	n+(1)	aCGH
*aif1::HYG* FLC^R16^	CPS111	>256	n+(1)	aCGH
*aif1::HYG* FLC^R16^	CPS114	>256	n+(1)	qPCR
*aif1::HYG* FLC^R16^	CPS126	>256	n+(1)	qPCR
*aif1::HYG* FLC^R16^	CPS128	>256	n+(1)	qPCR
*aif1::HYG* FLC^R16^	CPS135	>256	n+(1)	qPCR
*aif1::NAT AIF1-NEO* FLC^R6^	CPS177	16	n	aCGH
*aif1::NAT AIF1-NEO* FLC^R6^	CPS178	48	n+(1)	aCGH
*aif1::NAT AIF1-NEO* FLC^R6^	CPS179	48	n+(1)	aCGH
*aif1::NAT AIF1-NEO* FLC^R6^	CPS183	8	n	qPCR
*aif1::NAT AIF1-NEO* FLC^R6^	CPS184	8	n	qPCR
*aif1::NAT AIF1-NEO* FLC^R6^	CPS185	24	n	qPCR
*aif1::NAT AIF1-NEO* FLC^R6^	CPS189	16	n	qPCR
*aif1::NAT AIF1-NEO* FLC^R6^	CPS190	8	n	qPCR
*aif1::NAT AIF1-NEO* FLC^R6^	CPS193	16	n	qPCR
*aif1::NAT AIF1-NEO* FLC^R6^	CPS194	32	n	qPCR
RCT 17 FLC^R16^	CPS104	>256	n+(1)	aCGH
RCT 17 FLC^R16^	CPS136	64	n+(1)	qPCR
RCT 17 FLC^R16^	CPS141	>256	n+(1)	qPCR
RCT 17 FLC^R16^	CPS142	>256	n+(1)	qPCR
RCT 17 FLC^R16^	CPS152	>256	n+(1)	aCGH
RCT 17 FLC^R16^	CPS153	>256	n+(1)	qPCR
RCT 17 FLC^R16^	CPS157	>256	n+(1)	aCGH
RCT 17 FLC^R16^	CPS158	>256	n+(1)	qPCR
RCT 17 FLC^R16^	CPS159	>256	n+(1)	qPCR
RCT 17 FLC^R16^	CPS160	192	n+(1)	qPCR
RCT 17 *AIF1-NEO* FLC^R16^	CPS180	32	n	aCGH
RCT 17 *AIF1-NEO* FLC^R16^	CPS181	96	n+(1)	aCGH
RCT 17 *AIF1-NEO* FLC^R16^	CPS182	64	n+(1)	aCGH
RCT 17 *AIF1-NEO* FLC^R16^	CPS186	32	n	qPCR
RCT 17 *AIF1-NEO* FLC^R16^	CPS187	16	n	qPCR
RCT 17 *AIF1-NEO* FLC^R16^	CPS188	32	n	qPCR
RCT 17 *AIF1-NEO* FLC^R16^	CPS191	16	n	qPCR
RCT 17 *AIF1-NEO* FLC^R16^	CPS192	32	n	qPCR
RCT 17 *AIF1-NEO* FLC^R16^	CPS195	32	n	qPCR
RCT 17 *AIF1-NEO* FLC^R16^	CPS196	32	n	qPCR

Data provided in Figures 2, 5, S3B, and S4.

*FLC^R^ corresponds to MIC indicated in Table 1.

**Numbers in parentheses represent disomic chromosomes; segmental duplications are indicated as “dup”.

In the wild-type H99 background, four out of eight colonies (50%) isolated from Etest halos had MICs greater than 48 µg/ml for FLC and also had Chr1 disomy (Table 3, Figure 2B). The four colonies that presented MICs less than 48 µg/ml were euploid (Table 3). Resistant colonies isolated from a second round of Etests presented higher MICs than the original four strains, and in all cases Chr1 was duplicated (Table 3, Figure 2C). Notably, all *aif1* FLC^R^ colonies (100%) isolated directly from Etest experiments became completely resistant to FLC and also had whole or partial Chr1 disomy (Table 3). From our aCGH analysis, it was clear that some FLC^R^ colonies in the *aif1* background had whole or partial chromosome duplications in addition to Chr1, which were not observed for the H99 wild-type background (Figure 2D-E, Table 3, Figure S4).

A complemented strain, in which the *AIF1* gene with its native promoter and terminator was ectopically integrated into the genome, behaved like the wild-type strain in Etest assays (Figure 2A) and FLC fluctuation analysis (Table 2). Only two out of ten colonies (25%) isolated from Etest halos had MICs greater than 48 µg/ml for FLC and also had Chr1 disomy (Table 3), while the remaining colonies were euploid. In conclusion, aneuploidy and FLC resistance emerge at higher frequency in the absence of Aif1.

### Aneuploid isolates are stable and do not show decreased stress resistance

FLC resistance acquired through Chr1 disomy was reported to be lost during passage in drug-free media, and the clones returned to their original euploid state [33], [35]. To test if FLC^R^ aneuploid isolates would lose their adaptive phenotype upon removal of the selective pressure imposed by the drug, we chose to test two isolates with Chr1 disomy [n+(1)] and FLC resistance in wild-type H99, the *aif1* mutant, and the *aif1*+*AIF1* complemented strain. Upon growth in FLC-free liquid medium for about 20 generations, the isolates in the wild-type (CPS106 and CPS149) and complemented (CPS178 and CPS179) backgrounds returned to the native heteroresistant phenotype (Table 4). However, the isolates in the *aif1* background (CPS18 and CPS24) did not lose the FLC resistance phenotype during nonselective growth (Table 4). These results indicate that the aneuploid FLC^R^ cells are stabilized and tolerated in the absence of Aif1.

**Table 4:** Reversion rates of resistance to FLC after 20 generations

Strain	Background	FLC^R^ reversion rate (%)*
CPS106	WT	96.0±6.9
CPS149	WT	96.3±2.5
CPS18	*aif1*	9.0±5.9
CPS24	*aif1*	3.0±0.8
CPS178	*aif1*+*AIF1*	81.7±2.9
CPS179	*aif1*+*AIF1*	76.3±3.5
CPS104	RCT 17	7.7±4.7
CPS157	RCT 17	0.0±0.0
CPS181	RCT 17+*AIF1*	75.7±1.3
CPS182	RCT 17+AIF1	72.3±4.5

*Reversion rate was calculated based on three independent experiments and values represent averages ± standard deviation.

**Figure 3:**
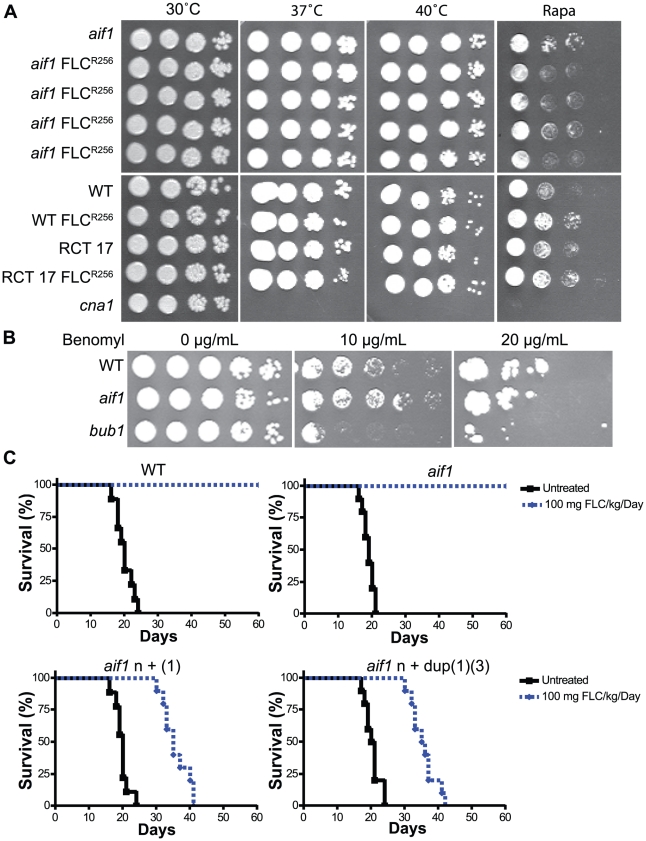
Fluconazole resistant aneuploid clones are stable and virulent. A . Aneuploid FLC^R^ clones are not temperature sensitive or more sensitive to the inhibitor rapamycin. Strains from the top: CPS3, CPS18, CPS24, CPS37, CPS48, H99, CPS106, RCT 17, CPS104, and KK8 (used as a control for temperature-sensitivity). **B**. The *aif1* mutant has a functional mitotic checkpoint in response to the indicated concentrations of the microtubule-destabilizing drug benomyl. **C**. FLC^R^ clones are more resistant to FLC treatment than wild-type and *aif1* mutants in murine cryptococcal infection model. Mice were infected intranasally with strains H99, CPS3, CPS18, and CPS24 and treated with 100 mg/kg/day of FLC.

Aneuploid *S. cerevisiae* strains bearing an extra copy of various chromosomes displayed decreased resistance to stress, such as increased sensitivity to high temperatures and to protein synthesis inhibitors [37]. We tested whether the FLC^R^ aneuploid isolates that we isolated had a similar phenotype. As shown in Figure 3A, none of the tested aneuploid strains (including isolates of different strain backgrounds and containing various chromosomal amplifications) showed increased sensitivity to high temperatures or to the protein synthesis inhibitor rapamycin. The only difference that we noticed in the aneuploid strains compared with euploid strains was the presence of elongated and larger cells (data not shown), which was also observed by Sionov et al. [35]. Because there was no difference between aneuploid strains in the *AIF1* and *aif1* backgrounds, we did not further investigate this phenotypic characteristic.

### Increased aneuploidy in *aif1* mutant is not due to a defect in mitotic checkpoint

We considered that the increased frequency of FLC^R^ subpopulations in the *aif1* mutant could be explained by two possible mechanisms. First, it is possible that Aif1-mediated ALCD would eliminate aneuploid cells from the population, and in the absence of Aif1, aneuploid cells persist within the population and emerge as FLC^R^ isolates. Another hypothesis is that Aif1 has non-apoptotic functions related to proper chromosomal segregation.

To test if *aif1* has mitotic spindle checkpoint defects, we examined its sensitivity to benomyl. In *S. cerevisiae*, hypersensitivity to the microtubule-destabilizing drug benomyl of “budding uninhibited by benzimidazole” (*bub*) mutants correlates with their mitotic checkpoint defect [38], [39]. As shown in Figure 3B, the *aif1* mutant did not exhibit sensitivity to benomyl. To eliminate the possibility that benomyl is simply not taken up by *C. neoformans*, we deleted the gene CNAG_03184, which was the first reciprocal BLASTp hit with *S. cerevisiae* Bub1, in the H99 background. As expected, the *bub1* mutant showed an increased sensitivity to benomyl, indicating that this drug is active in *C. neoformans*. In conclusion, because *aif1* has an intact mitotic spindle checkpoint, we propose that Aif1-mediated ALCD eliminates aneuploid cells from the population.

### Aneuploid isolates are resistant to fluconazole treatment in vivo

*C. neoformans* strains with FLC resistance levels greater than 32 µg/ml were previously reported to have higher virulence in mice [33]. To test if that would also be the case for isolates in the *aif1* mutant background, we tested the virulence of two strains completely resistant to FLC using an inhalation murine model of cryptococcosis. Mice were intranasally infected with the indicated strains, and their survival was monitored and plotted against time. The *aif1* mutant has a virulence level similar to wild-type (see below). As shown in Figure 3C, FLC^R^ strains CPS18 [FLC^R256^ n+(1)] and CPS24 [FLC^R256^ n+dup(1)(3)] showed similar virulence to the H99 strain (untreated cohort of animals; p-values of 0.5739 and 0.5252, respectively).

Next, we tested the efficacy of antifungal therapy when infection is caused by FLC^R^ strains. Treatment with 20 mg/kg/day of FLC was initiated 24 hours after infection and was continued for 14 days, as described by [33]. As shown in Figure S5, treatment with 20 mg/kg/day of FLC for 14 days modestly prolonged the survival of mice inoculated with wild-type H99 (p-value of 0.0043) and *aif1* mutant (p-value of 0.0023) strains, but it did not prolong the survival of cohorts inoculated with the CPS18 (p-value of 0.1443) and CPS24 (p-value of 0.2237) resistant isolates. In a second set of experiments, we evaluated the efficacy of a higher FLC dosage, and animals received 100 mg/kg/day of FLC, without treatment interruption. All mice inoculated with wild-type H99 and *aif1* mutant strains and treated with 100 mg/kg/day of FLC survived for 60 days (p-values <0.0001). On the other hand, while treatment with 100 mg/kg/day of FLC prolonged the survival of mice inoculated with the CPS18 and CPS24 resistant isolates (p-values <0.0001), 100% mortality was observed in these cohorts (Figure 3C).

**Figure 4:**
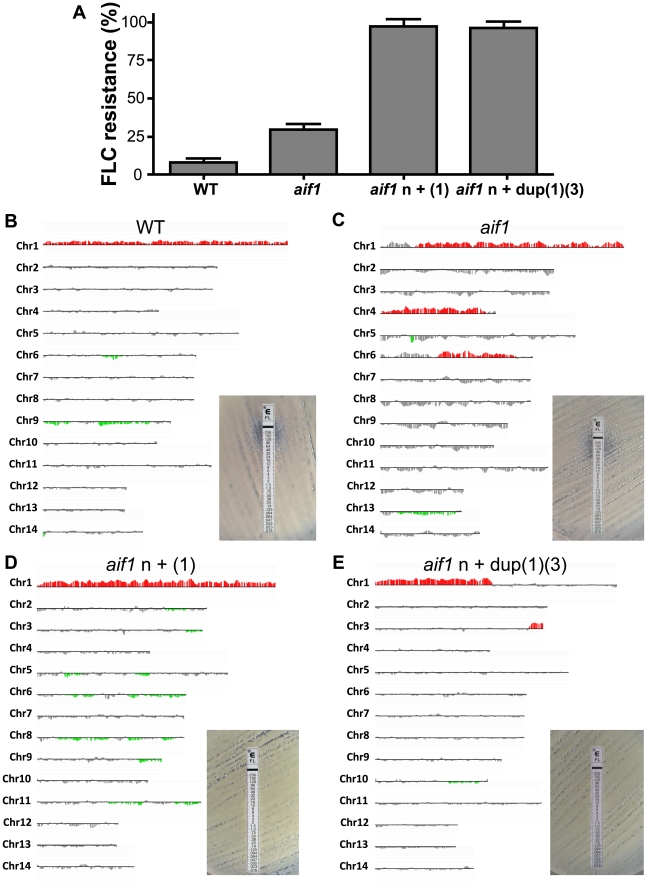
Strains recovered from treated mice are fluconazole resistant and aneuploid. A . Percentage of FLC^R^ colonies isolated from lungs and brains of 3 mice treated with FLC. *, ** and *** indicates significance at p<0.05, 0.01 and 0.001, respectively. **B-E**. aCGH analysis of FLC^R^ colonies recovered from mice infected intranasally with strains H99 (**B**, CPS129), CPS3 (**C**, CPS134), CPS18 (**D**, CPS145), and CPS24 (**E**, CPS146).

Additionally, we examined if resistant strains would appear during FLC treatment and if aneuploidy would be stable with in vivo passage. We randomly selected three mice treated with 100 mg/kg/day of FLC for each strain. For CPS18 and CPS24 cohorts, the mice were sampled post-mortem between 30 and 35 days post-infection. For H99 and *aif1* cohorts, we used asymptomatic mice sacrificed on day 60, when the experiment was terminated. Colonies recovered on YPD medium from lungs and brains were tested for resistance to FLC. About 8% of wild-type and 29% of *aif1* colonies developed FLC^R^ in vivo during treatment, while more than 96% of CPS18 and CPS24 colonies retained their initial FLC resistance acquired in vitro (Figure 4A). The aCGH analysis of a wild-type H99 FLC^R^ colony (CPS129) selected randomly showed that this isolate acquired Chr1 disomy (Figure 4B). Ploidy analysis by FACS indicated that CPS129 was diploid [2n+(1), data not shown]. Increased ploidy has recently been described in *C. neoformans* to be associated with the formation of giant yeast cells in infected animals [40], [41]. The aCGH analysis of an *aif1* FLC^R^ colony (CPS134) showed duplication of chromosomes 1, 4, and 6 (Figure 4C), while FLC^R^ colonies in the CPS18 (CPS145) and CPS24 (CPS146) backgrounds maintained their initial state of aneuploidy (Figure 4D and 4E). These in vivo results can be correlated to our in vitro experiments (Table 2 and Table 4), but it is clear that the interaction of *C. neoformans* with the murine host environment promotes genomic plasticity, which serves to generate new phenotypic adaptations in this pathogen with disease management consequences.

### Clinical isolate RCT 17 shows increased fluconazole resistance associated with aneuploidy and reduced *AIF1* expression

To evaluate and validate if defects in the apoptotic pathway of *C. neoformans* have any impact on azole resistance in the human clinical setting, we tested the susceptibility to FLC of seven clinical isolates taken directly from frozen cerebrospinal fluid (CSF) of individual patients with cryptococcal meningitis. Microevolution and phenotypic variability of *C. neoformans* isolates occurs in the laboratory through multiple in vitro passages (reviewed by [42]–[44]). Therefore, an important factor considered in our experimental design when testing clinical isolates was the use of colonies isolated directly from CSF. We also targeted initial clinical isolates, collected before the commencement of antifungal therapy. It is important to note that the RCT isolates originated from South Africa, an HIV endemic area, and FLC is sometimes used as a prophylactic antifungal treatment in HIV/AIDS patients, and thus we cannot completely rule out prior exposure to azoles in these isolates.

**Figure 5:**
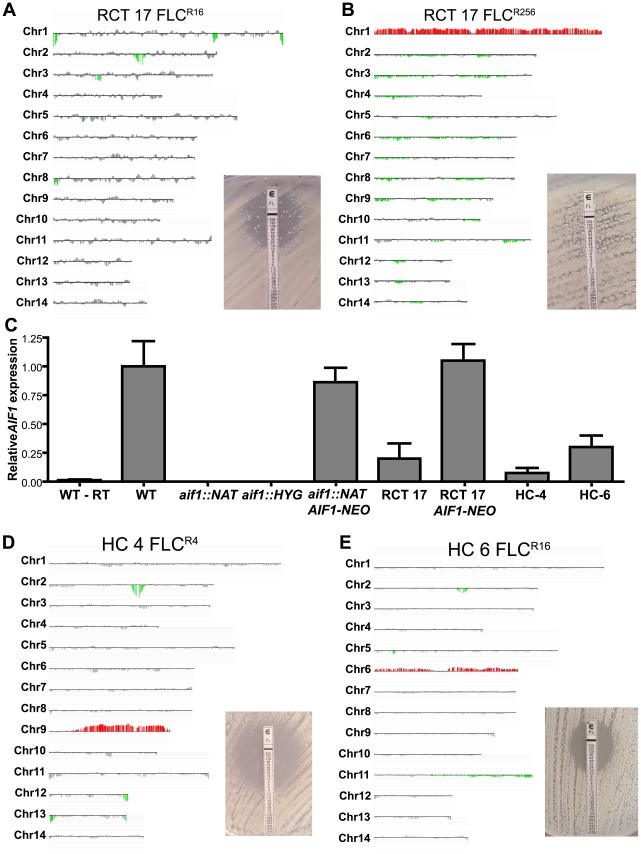
Detection of fluconazole resistance and aneuploidy in clinical isolates with *AIF1* deficiency. A . Clinical isolate RCT 17 shows a high frequency of FLC^R^ clone generation, a phenotype similar to that observed in the *aif1* mutant. **B**. aCGH analysis of a FLC^R^ colony isolated from RCT 17 on Etest assay showing Chr1 disomy (CPS104). **C**. qRT-PCR revealed that RCT 17, HC-4, and HC-6 have reduced *AIF1* expression. Strains were grown overnight in liquid YPD medium, and total RNA was collected and reverse-transcribed into cDNA (WT-RT represents control without reverse transcriptase). Relative expression levels represent mean ΔCt values normalized to *ACT1* expression levels and relative to expression values in the H99 wild-type strain (value arbitrarily set to 1). Bars represent standard deviation for triplicates. **D**. aCGH analysis of clinical isolate HC-4, showing partial duplication for chromosome 9. **E**. aCGH analysis of clinical isolate HC-6, showing partial duplication for chromosome 6. Vertical bars in red indicate amplifications, and in green deletions, that are statistically significant. Vertical bars in gray indicate no DNA copy number alteration or amplifications or deletions that did not meet the statistical settings.

As shown in Table 1, the susceptibility to FLC of the seven direct clinical isolates varied, with MICs ranging from 4 to >256 µg/ml. Specifically, isolate RCT 17 showed FLC^R^ colonies within the zone of inhibition around the FLC strip in Etest assays, similar to the *aif1* mutants (Figure 5A). Moreover, such colonies were completely resistant to FLC and exhibit Chr1 disomy (Figure 5B, and Table 3). RCT 17 showed a FLC resistance rate that was about 20 times higher than H99 and 4 times higher than the *aif1* strain (Table 2). The FLC^R^ isolates CPS104 and CPS157 in the RCT 17 background did not revert to FLC sensitivity during growth on FLC-free medium for 20 generations (Table 4), showing that aneuploid FLC^R^ cells are stable in the RCT17 background, as was observed for the *aif1* mutant.

Given the similarities between the clinical isolate RCT 17 and the *aif1* mutant, we hypothesized that the *AIF1* gene might be defective in this clinical isolate. Quantitative RT-PCR (qRT-PCR) revealed that *AIF1* expression is approximately 80% reduced in the RCT 17 isolate compared to the H99 strain (Figure 5C). DNA sequence analysis determined the presence of five nucleotide polymorphisms in the *AIF1* promoter and coding sequence from RCT 17 compared to the H99 (data not shown). Of note, the primer pair used for the qRT-PCR was designed to encompass the boundaries of exons 1 and 2, a region that has no polymorphisms in the RCT 17 isolate. Complementation of the RCT 17 strain with the *AIF1* gene and its native promoter and terminator restored normal *AIF1* expression levels (Figure 5C), reduced the FLC heteroresistance rate (Figure S6A and Table 2), and reversed the stability of the aneuploid Chr1 in the absence of FLC (Table 4), indicating that low *AIF1* expression levels are responsible for the RCT 17 *aif1*-like phenotypes.

Additionally, we evaluated *AIF1* expression in two other clinical isolates from the USA that were reported to be disomic for other chromosomes besides Chr1. Isolates HC-4 and HC-6 show partial duplication for chromosomes 9 and 6, respectively (Figure 5D and 5E and Hu and Kronstad et al., manuscript in preparation). *AIF1* expression is also reduced in isolates HC-4 and HC-6 (Figure 5C), indicating that downregulation of *AIF1* is not restricted to cryptococcal cells under strong selective drug pressure and might be a novel aneuploidy-tolerating mechanism.

### Apoptotic pathways are required for sporulation during mating in *C. neoformans*

During the genetic manipulations of our apoptotic mutants, we noticed that they displayed defective mating phenotypes. Mating in *C. neoformans* is initiated by fusion between α and **a** cells to produce dikaryotic filaments, and the process culminates in the formation of basidia decorated with four spore chains. Spores formed by mating represent infectious propagules for *C. neoformans*. Mating filament production was reduced but not abolished when *aif1*, *mca1 mca2*, and *aif1 mca1 mca2* independent mutants of opposite mating types were subjected to bilateral mutant crosses (Figure S7A). In unilateral crosses (mutant × wild-type), the *aif1*, *mca1 mca2*, and *aif1 mca1 mca2* mutants had delayed formation of filaments compared with a wild-type cross, but were still fertile (data not shown). Bilateral crosses of the *aif1 mca1 mca2* triple mutant showed more severe defects in filamentation, indicating that both Aif1 and the metacaspases have a role in *C. neoformans* mating. In confrontation assays between the *aif1*, *mca1 mca2*, and *aif1 mca1 mca2* mutants and a *crg1* mutant strain, which is hypersensitive to mating pheromone and produces abundant conjugation tubes when confronted with wild-type cells of the opposite mating type [45], no conjugation tubes were produced, indicating a decrease in pheromone secretion by the *aif1*, *mca1 mca2* and *aif1 mca1 mca2* mutants (Figure S7B).

Decreased hyphal growth during mating can be a result of defective fusion between α and **a** cells. To determine if this was the case in the *aif1* and metacaspase mutants, cell fusion assays using opposite mating type strains with dominant genetic markers in bilateral crosses were conducted. The formation of double-resistant diploids by cell fusion was quantified after 48 h of incubation on mating medium. Many diploid isolates were produced from the wild-type control cross compared with the bilateral mutant crosses (Figure S8). We also examined fusion in unilateral crosses between the *mca1 mca2* mutant and wild-type strains. The number of diploid isolates produced from these crosses was considerably lower than those with wild-type strains, demonstrating that one copy of each metacaspase gene is not sufficient for fusion (Figure S8). Hence, Aif1 and the metacaspases are required for the cell fusion step of mating in *C. neoformans*.

**Figure 6:**
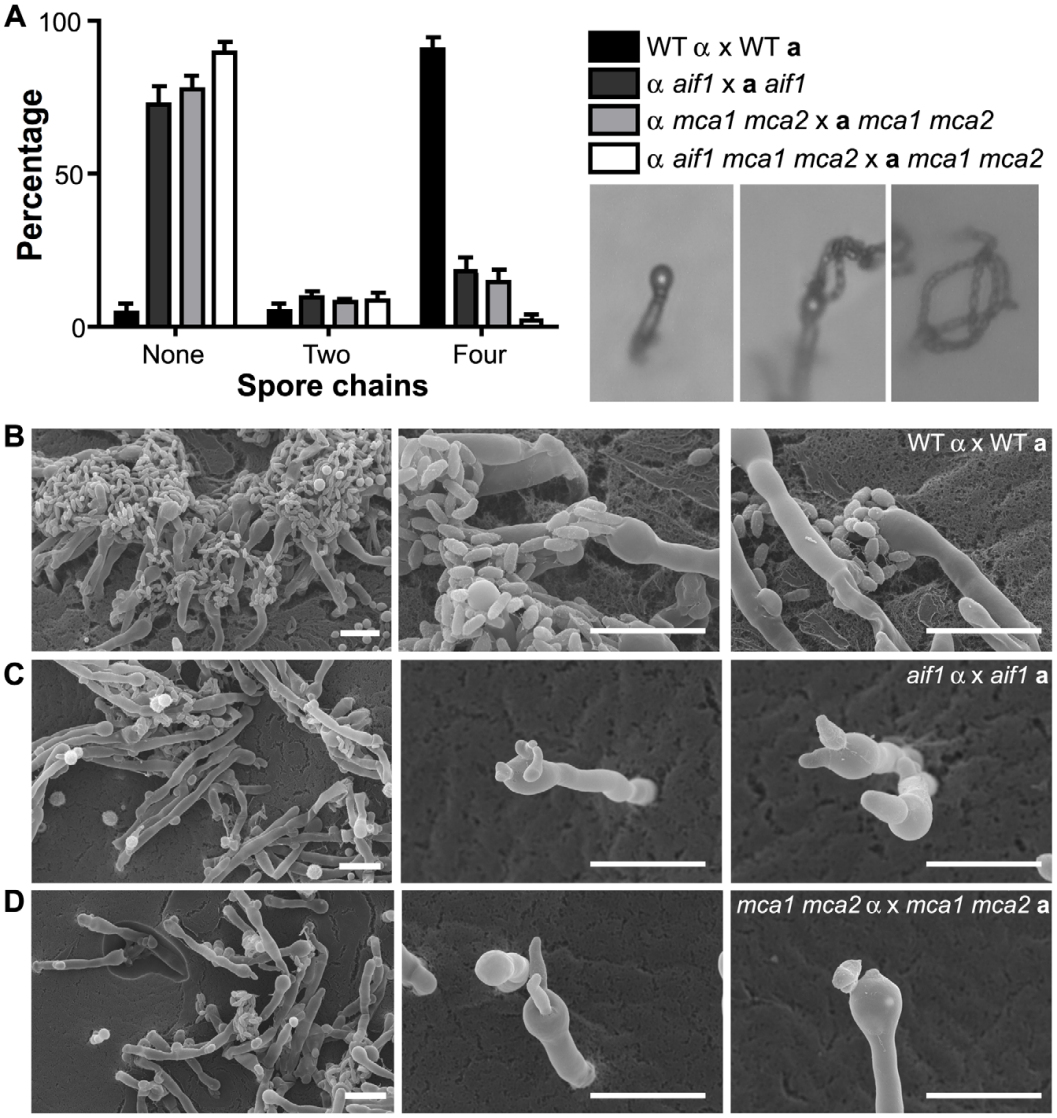
Apoptotic-defective mutants show defective basidiospore formation. A . Sporulation defects were observed in bilateral matings between α and **a** strains: H99 x KN99**a**; CPS3 x CPS15; YPH104 x CPS13; and CPS89 x CPS116. Basidia with zero, two or four spore chains formed after 14 days on MS media were counted using 40X microscopy (inset shows representative images). **B-D**. Scanning electron microscopy images of the wild-type H99 x KN99**a** (**B**), *aif1* x *aif1* mutant CPS3 x CPS15 (**C**), and *mca1 mca2* x *mca1 mca2* mutant YPH104 x CPS13 (**D**) crosses. Basidia and basidiospore chains are shown in greater detail in the middle and right panels. Bars, 10 µm.

Additionally, a significant reduction in basidiospore production was observed in bilateral mutant crosses (Figure 6A). The *aif1* and *mca1 mca2* mutants produce defective basidia with aberrant spore chains (Figure 6A-D), although these strains were not sterile, and we were able to isolate recombinant spores in which the mutations were recovered in the opposite mating type. Sporulation in the *aif1 mca1 mca2* bilateral mutant cross was impaired and most basidia produced few spores, in contrast to the four long spore chains observed in a wild-type cross (Figure 6). Basidiospore production defects were also observed in unilateral crosses between *aif1 mca1 mca2* mutant and wild-type strains (data not shown). We conclude that ALCD is required for the proper sporulation of *C. neoformans*.

### The *aif1* and metacaspase mutants show decreased competitive fitness but have no defects in virulence

**Figure 7:**
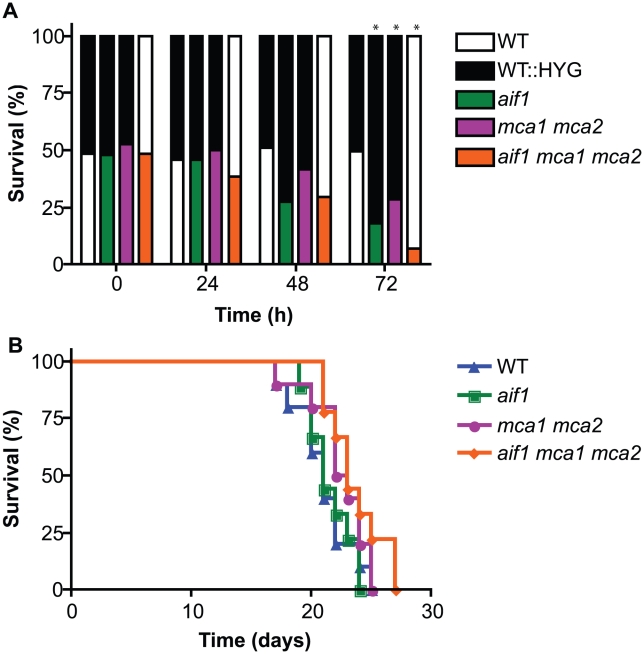
Apoptosis-like cell death is important for competitive fitness. A . Competition assays showing that ALCD is important to population fitness in vitro. Strains H99 and CPS76 (H99 HYG), H99 and CPS3, H99 and YPH104, and H99 and CPS89 were paired at the same cell density and each mixture was inoculated into YPD liquid medium at a final density of 8×10^4^ cells/ml and incubated at 30°C for the indicated times. An aliquot of each culture was plated on YPD solid medium, and incubated for 2 days before it was replica plated onto YPD, YPD-HYG, YPD-NAT, and YPD-NEO media. The ratios of survival for each strain at 0, 24, 48, and 72 h are shown for one representative of three experiments and *indicates significance at p<0.05. **B**. Virulence of the apoptotic defective strains in the murine model shows no significant difference to wild-type. Mice were infected intranasally with strains H99, CPS3, YPH104, and CPS89.

Apoptosis plays an essential role in maintaining homeostasis in multicellular organisms by eliminating permanently damaged cells. In the unicellular yeast *S. cerevisiae*, ALCD has been proposed to eliminate the less fit individuals from a monoclonal population of cells, thereby improving the chance of survival of the fitter clones in the genetic pool [46], [47]. To determine whether the apoptotic pathways are relevant to the ability of *C. neoformans* populations to compete for resources in a common environment, the fitness of apoptotic null mutants was measured in pair-wise growth competition assays with wild-type. The same amount of cells from wild-type and apoptotic null mutant strains grown to exponential phase were mixed in liquid YPD medium and incubated at 30°C with agitation. Each strain was identified based on differing genetic markers, including a control sample using two wild-type strains. To reduce cell fusion and mating during the experiment, all strains used were of the α mating type. Aliquots of each culture were taken at the indicated times and plated on selective media to calculate the survival ratios. As shown in Figure 7A, the apoptotic defective mutants were outcompeted by the wild-type cells. Because the *aif1*, *mca1 mca2*, and *aif1 mca1 mca2* mutants had similar division rates as wild-type in liquid YPD medium at 30°C during solo culture (Figure S9), we eliminated the possibility that the wild-type cells were simply more fit to begin with, and thus concluded that wild-type cells exhibit enhanced fitness during co-culture with apoptotic mutant cells.

To determine whether the apoptotic null mutants had any growth defect in the host environment, we performed virulence assays in a murine inhalation model of cryptococcosis. Mice were intranasally infected with wild-type and mutant strains, and their survival was monitored and plotted against time (Figure 7B). Compared to the wild-type strain, the apoptotic defective mutants showed no significant difference in virulence (Figure 7B), fungal burden, or organ dissemination pattern (data not shown). Therefore, inactivation of ALCD had no apparent impact on the virulence of *C. neoformans* in these tested conditions.

## Discussion

**Figure 8:**
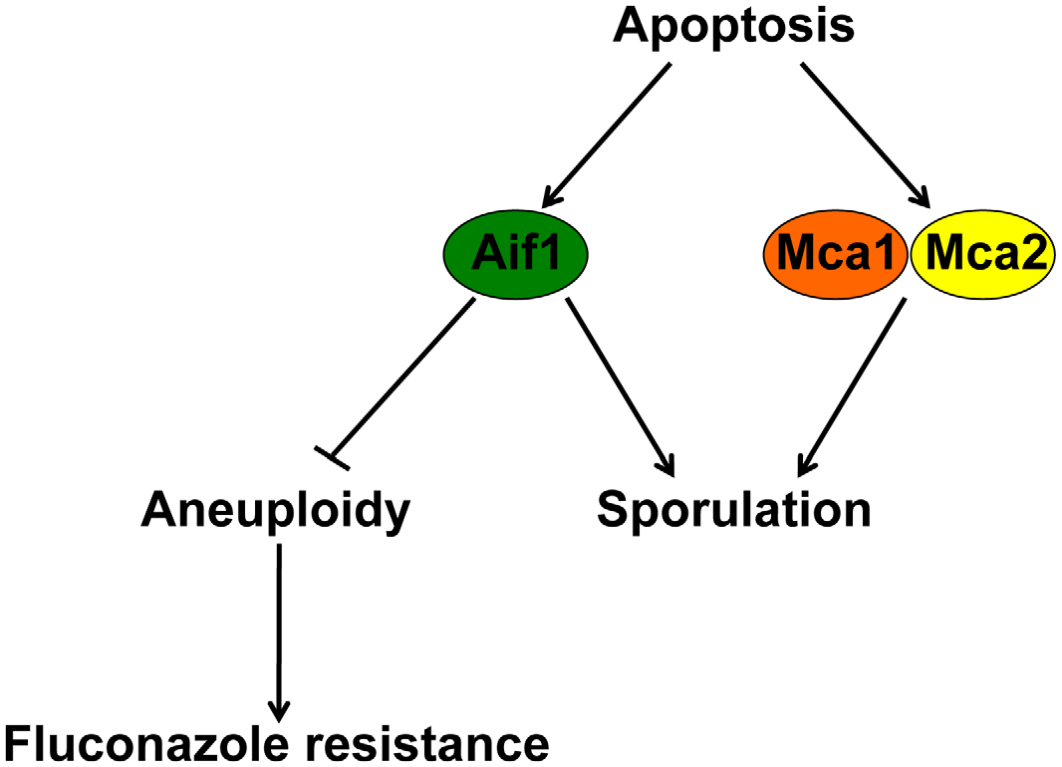
Mechanisms controlling ALCD, mating, and aneuploidy. Both Aif1 and the metacaspases are required for sporulation in *C. neoformans*. The Aif1-mediated ALCD eliminates aneuploid cells from the population and contributes to the emergence of FLC^R^ cells.

Apoptosis was long thought to be restricted to metazoans, but a growing body of evidence has established that it appears to be a common feature of diverse organisms. During the past decade, ALCD has been described in the majority of phylogenetic lineages including eubacteria, protists, plants, and fungi [11], [48], [49]. While the body of evidence supporting apoptotic fungal cell death is growing, the molecular mechanisms underlying fungal apoptotic pathways and their physiological relevance are poorly understood. Here, we show that Aif1 and metacaspases are independently required for apoptosis in the human pathogen *C. neoformans*. While both Aif1 and the metacaspases are important for sexual development, only Aif1 is necessary for ploidy maintenance (Figure 8, further discussed below).

We found that the *aif1*, *mca1 mca2*, and *aif1 mca1 mca2* of *C*. *neoformans* have impaired sexual reproduction with a consequent reduction in formation of spores as infectious propagules (Figure 6). Similarly, the formation of ascospores in *Podospora anserina* was severely impaired in mutants with defective metacaspases [50] and the apoptosis-inducing factor homolog Amid2 [51]. Apoptotic markers, such as positive TUNEL reactions, were observed during sporulation in the basidiomycete *Coprinopsis cinerea* [52]. Furthermore, analysis of sporulation-defective mutants revealed the existence of a state in which, although meiosis was successfully completed, basidiospore formation was impaired due to lack of apoptosis [52].

Wild-type *C. neoformans* cells were observed to outgrow the *aif1*, *mca1 mca2*, and *aif1 mca1 mca2* mutants in competitive growth assays. Likewise, in *S. cerevisiae*, the metacaspase *yca1* and *aif1* deletion mutants also show a decreased competitive fitness phenotype [47], [53]. ALCD was proposed to contribute to the fitness and adaptability of yeast populations by removing damaged or less fit clones. The fact that the *aif1*, *mca1 mca2*, and *aif1 mca1 mca2* mutants cannot outcompete wild-type cells for resources in a common environment might explain, at least partially, why natural selection has preserved the apoptotic pathways in unicellular yeasts. It is also reasonable to consider that yeast apoptotic proteins may also participate in vital non-death functions. In support of this hypothesis, Yca1 was shown to have non-death roles in the timing of the yeast cell cycle [54] and the removal of insoluble protein aggregates [55].

It is interesting to highlight that the evolutionary implications of removing ALCD depend on selective pressure. For example, under conditions to which wild-type cells are well adapted, the *aif1* mutant is not able to outcompete for environmental resources. On the other hand, under a strong selective pressure such as drug treatment, *AIF1* inactivation promotes the stabilization of aneuploidy and thus rapid adaptation to the stress of FLC. Such observations can be compared to the known roles of apoptosis in multicellular eukaryotes; while apoptosis effectively promotes tissue homeostasis, its inactivation promotes tumorigenesis and the emergence of drug-resistant cancer cells.

### Links between Aif1, aneuploidy, and the emergence of drug resistance

Alteration in gene copy number is a major mechanism for environmental adaptation of asexual yeast populations. In *S. cerevisiae*, aneuploidy was shown to confer improved growth and fitness advantages under stressful conditions [37], [56], [57]. Aneuploidy was also associated with FLC resistance in *Candida* species [58]–[61]. In *C. albicans*, a specific segmental aneuploidy, isochromosome 5L [i(5L)], is commonly found in FLC^R^ isolates, and the loss of i(5L) is correlated with reduced FLC resistance [58]. Gain of i(5L), which is comprised of two identical chromosome 5 left arms flanking a centromere, amplifies two genes that contribute additively and independently to FLC resistance: *ERG11* (the target of FLC) and *TAC1* (a transcription factor that activates expression of the drug efflux pumps *CDR1* and *CDR2* [59]).

The acquisition of aneuploidy was recently reported in strains of *C. neoformans* that display heteroresistance to FLC [35]. Heteroresistance in *C. neoformans* is defined as heterogeneous FLC susceptibility within a population with resistant subpopulations being able to adapt to higher concentrations of the drug in a stepwise and reversible manner [31]–[33]. Heteroresistance is considered different from trailing phenomena or incomplete growth inhibition by azoles observed in *Candida* species. Trailing growth can cause the MICs of azoles for some isolates to be low (susceptible) after 24 h of growth but much higher (resistant) after 48 h [62]. The relevance of such discordant interpretive categories at the two time points is as yet unclear, but current evidence suggests that the lower MIC correlates most closely with the outcome in vivo [62]. Bacterial populations were reported to produce persister cells that neither grow nor die in the presence of microbicidal antibiotics. Persisters are largely responsible for high levels of biofilm tolerance to antimicrobials, but do not exhibit an increased MIC. *C. albicans* persister cells that survived killing by AMB were detected only in biofilms and not in exponentially growing or stationary-phase planktonic populations [63].

Even though we used Etest assays to isolate FLC^R^ colonies in this study, our results support previous findings in which the H99 strain exposed to FLC in liquid cultures became resistant in a step-wise pattern to increasing concentrations of the drug after acquisition of specific chromosomal duplications [33], [35]. In our experiments, Chr1 disomy was observed in H99 derivatives that became resistant to more than 48 µg/ml of FLC and was more common on second Etest passages, while disruption of *aif1* stimulated the selection of completely FLC^R^ (MIC >256 µg/ml) subpopulations. Amplification of chromosomes other than Chr1, particularly Chr4, was observed in *aif1* derivatives, but not in the H99 or *AIF1* complemented derivatives isolated in Etest experiments (Table 3). In the *aif1* derivatives, segmental chromosomal duplications were also observed; isolate CPS24 had the left arm of Chr1 duplicated, which harbors *ERG11*, and the end of the right arm of chromosome 3 (Figure 2E and Figure S3A). *C. gattii* isolates resistant to 64 µg/ml of FLC showed different patterns of chromosomal amplifications, including disomy and segmental duplication of several chromosomes besides Chr1 [64]. Clinical isolate RCT 52 had a FLC MIC >256 µg/ml (Table 1) but did not show any chromosomal amplification (data not shown), indicating that not all FLC^R^ isolates are aneuploid. These findings emphasize the existence of FLC resistance mechanisms that are independent of Chr1 disomy.

After about 20 generations of non-selective growth, more than 90% of FLC^R^ colonies in the H99 background and 75% in the complemented strain returned to being FLC sensitive, while less than 10% in the *aif1* background lost resistance to FLC upon removal of the drug pressure (Table 4). The lack of benomyl sensitivity in the *aif1* mutant excludes the possibility that the observed increased aneuploidy in this strain is a result of a defective mitotic spindle checkpoint. Our results show that the lack of a functional Aif1 was able to stabilize the aneuploid state selected by FLC treatment, and we propose that *aif1* is an aneuploidy-tolerating mutation in *C. neoformans*.

Torres et al. [56] described the existence of aneuploidy-tolerating mutations in yeast, such as the deubiquitinating enzyme Ubp6, which improves growth rates in aneuploid yeast strains by attenuating the protein stoichiometry imbalances caused by chromosomal amplifications. We did not observe that FLC^R^ aneuploid strains display an increased sensitivity to stresses, such as reported in *S. cerevisiae* by Torres et al. [37]. The results shown in Figure 3A suggest that aneuploidy does not inevitably result in decreased resistance to stress, and are in agreement with a recent study by Pavelka et al. [57], which showed that aneuploidy directly confers phenotypic variation that can result in a growth advantage under stress conditions that are suboptimal for euploid cells. The conflicting results in *S. cerevisiae* were explained by the different methods used to generate the aneuploid strains; while Torres et al. [37] constructed their aneuploid strains through selection with a combination of drug and nutrient markers, Pavelka et al. [57] generated aneuploid strains through random meiotic segregation, suggesting an impact of both karyotype and continuous selection with drugs and nutrient markers in the phenotypic variation conferred by aneuploidy [57]. Interestingly, as in Pavelka et al. [57], we observed that certain aneuploid strains were able to grow better in the presence of drugs such FLC and rapamycin (strain WT FLC^R256^, Figure 3A).

Rancati et al. [65] reported that *S. cerevisiae* cells lacking *MYO1*, which encodes the only myosin-II normally required for cytokinesis, rapidly acquired aneuploidy that led to specific changes in the transcriptome that restored growth and cytokinesis. Therefore, one possibility is that *aif1* mutants have more stable Chr1 aneuploidy because genes present on Chr1L might complement Aif1 functions. It should be noted that no growth defect (besides the decreased competitiveness with wild-type) was observed for *aif1* mutant strains, and that Chr1 aneuploidy did not appear to improve *aif1* mutant strains growth. We speculate that apoptosis orchestrated by Aif1 eliminates aneuploid cells from the population.

Why didn't the metacaspase mutants have an increased rate of aneuploidy and consequent resistance to FLC? Based on the fact that Aif1 and the metacaspases are independently required for apoptosis in *C. neoformans*, it is possible to speculate that the elimination of aneuploid cells from the population is regulated by a caspase-independent apoptotic pathway. In yeast, the Yca1 caspase-like protease participates in approximately 40% of the investigated cell death scenarios, while Aif1 and endonuclase G execute caspase-independent cell death [66]. It is also possible that ploidy maintenance is a non-death function of Aif1.

FLC^R^ colonies isolated from the H99 and *aif1* strains during our in vivo experiments showed more complex patterns of ploidy increase and amplifications (Figure 4B and 4C). The interaction of *C. neoformans* with mammalian hosts has been shown to promote genomic plasticity in this pathogen (reviewed by [67]). Comparative hybridization studies characterized disomy for chromosome 13 in two independent clinical isolates [68]. Additionally, the formation of giant cells with diameters up to 100 µm was observed during murine cryptococcal infection [40], [41]. These giant yeast cells were found mainly in lung tissue and were polyploid and uninucleate, suggesting that they arise from DNA endoreplication without concomitant cell division. It will be interesting to investigate if ALCD is also important in ploidy increase and the formation of giant yeast cells. In preliminary analysis of FLC Etests using a diploid wild-type strain, we did not observe an increase in heteroresistance while a diploid *aif1* strain still showed increased heteroresistance (Figure S6B).

In our in vivo virulence experiments, we evaluated the efficacy of FLC therapy for wild-type and FLC^R^ strains using a murine model of cryptococcal meningitis. Treatment with 100 mg/kg/day of FLC had antifungal activity against H99 and *aif1* strains (but did not clear infection in the brains of the animals) and doubled the survival time of mice infected with FLC^R^ strains. Pharmacodynamic studies, using the area under the concentration-time curve (AUC), showed that FLC therapy in doses of 100 mg/kg/day given to mice mimics doses of 400 mg/day given to humans [69]. Therefore, the use of higher dosages of FLC might be beneficial in patients, and may help to reduce morbidity and mortality due to cryptococcal meningitis in Africa and other resource-limited regions.

We found that RCT 17, one of seven direct clinical isolates, had increased heteroresistance to FLC, similar to the heteroresistance observed in the *aif1* mutants (Table 1, Figure 5A). Furthermore, FLC^R^ colonies isolated from the RCT 17 background also became completely resistant to FLC through the acquisition of aneuploidy (Figure 5B, and Table 3) and did not revert to being FLC-sensitive during growth on FLC-free medium for 20 generations (Table 4). *AIF1* expression was shown to be downregulated in RCT 17 by qRT-PCR (Figure 5C), and complementation with *AIF1* with its native promoter and terminator recovered normal expression levels and reverted the increased heteroresistance to FLC (Table 2 and Table 3). *AIF1* expression was also reduced in the HC-4 and HC-6 clinical isolates (Figure 5C), which show disomies for chromosomes other than Chr1 and are not resistant to FLC (Table 1, Figure 5D and 5E). Therefore, we propose that the inactivation of *AIF1* is a novel aneuploidy-tolerating mechanism in fungi that might not be restricted to cells under strong selective FLC drug pressure. Future investigations will test the mechanisms that result in *AIF1* downregulation.

Our results suggest that triggering fungal ALCD should be further investigated as a new antifungal approach. Potential antifungal targets include those that can help another antifungal agent by blocking resistance mechanism(s) in vivo used by the microorganisms. This principle has previously been demonstrated by beta-lactamase inhibitors in antibacterial combination drugs [70] but has yet to be applied in therapies including new antifungal agents. An *AIF1*-inducing agent could have a profound effect on the ability of *C. neoformans* to become resistant in vivo to FLC, and the addition of such inducer to the antifungal regimen could make FLC fungicidal in the host.

In conclusion, our results bring to light the importance of the Aif1 in maintaining the euploidy of yeast populations, possibly through the elimination of aneuploid cells via apoptosis. Inactivation of *AIF1* might increase genomic plasticity of *C*. *neoformans* during infection and promote the generation of phenotypic adaptations such as new virulence traits and antifungal drug resistance.

## Materials and Methods

### Ethics statement

This study was carried out in strict accordance with The National Research Council's Guide for the Care and Use of Laboratory Animals, Public Health Service Policy on Humane Care and Use of Laboratory Animals, and AAALAC accreditation guidelines. The protocol was approved by the Duke University and Duke University Medical Center Institutional Animal Care and Use Committee (protocol number: A266–08–10). All efforts were made to minimize suffering. The clinical isolates were collected and stored in the Duke Infectious Disease Repository under an IRB-approved protocol entitled “Database and specimen repository for infectious diseases-related studies” (#CR3_Pro00005314). The specimens were de-identified discarded samples collected as part of routine clinical practice and were exempt from patient written informed consent.

### Strains and growth conditions

*C. neoformans* strains used in this study are listed in Table S1. YL99**a** strain was obtained by an extra round of H99 and KN99**a** backcrossing. The tested clinical isolates were taken directly from the CSF of patients with cryptococcal meningitis from USA (HC isolates, Duke patients) and from South Africa (RCT isolates, obtained from Tihana Bicanic and Tom Harrison). The indicated mating types were determined by crosses and confirmed with PCR, and the serotyping of clinical isolates was determined by PCR using *STE20* mating-type- and serotype-specific primer pairs [71]. Strains were maintained in −80°C glycerol stocks and grown on YPD medium (1% yeast extract, 2% Bacto Peptone, and 2% dextrose). To perform mating assays, cells of opposite mating type were mixed in water, spotted on 5% V8 juice agar medium (pH 5.0) or Murashige and Skoog (MS) medium minus sucrose and incubated at room temperature in the dark [72]–[75]. For spot assays, cultures grown in liquid YPD were diluted to 2×10^6^ cells/ml and serially diluted tenfold. Then, 5 µl of each culture was spotted onto YPD or YPD plus 2 mM H_2_O_2_ (Sigma) or YPD plus 20 ng/ml rapamycin (LC Laboratories) plates and incubated at the indicated temperatures. For benomyl sensitivity assays, 5 µl of fivefold serial dilutions were spotted onto YPD plates with DMSO (control) or with 10 or 20 µg/ml benomyl (Sigma). Growth differences were imaged following incubation of the plates for 72 h.

### Generation of gene disruption and complemented strains

Strains with gene disruptions were generated using an overlap PCR approach and biolistic transformation in serotype A congenic strains H99 and KN99**a** as previously described [76]. For gene deletions, the 5' and 3' flanking regions of apoptotic genes and *BUB1* were amplified from H99 genomic DNA and the dominant selectable markers NAT, NEO, or HYG were amplified with the universal M13F and M13R primers from plasmids pJAF13, pJAF12 and pJAF15 respectively. A second overlap PCR amplified a full-length deletion cassette containing the three previous fragments. The products of overlap PCR were purified, precipitated onto gold microcarrier beads (0.6 mm, Bio-Rad), and the H99 strain was biolistically transformed [5], [26]. Stable transformants were selected for resistance to nourseothricin (100 µg/ml), G418 (100 µg/ml), or hygromycin B (300 µg/ml), screened by PCR, and confirmed by Southern blot. The α *mca1* mutant background was used for disruption of *MCA2* by biolistic transformation. The resulting α *mca1 mca2* mutant was crossed to strain KN99**a** to generate single and double mutants with opposite mating types (see Table S1). The *aif1 mca1 mca2* triple mutants are progeny obtained from the cross of α *aif1*with **a** *mca1 mca2* (see Table S1). For complementation, an overlap PCR product with the NEO marker and the wild-type *AIF1* gene containing its native promoter and terminator from strain H99 was generated. The PCR product was biolistically transformed into *aif1* mutant and RCT17 backgrounds. Transformants were selected for resistance to G418 (100 µg/ml) and confirmed by PCR. The sequences of primers used are listed in Table S2.

### TUNEL assay

TdT-mediated dUTP nick end labeling (TUNEL) assays were performed as described previously [5], [26] with the following modifications. Log-phase cultures were diluted to an OD_600_ of 0.1 and treated with 2 mM H_2_O_2_ (Sigma) for 3 h at 37°C. Cells were fixed for 1 h at room temperature with 3.7% formaldehyde (Sigma) and washed with PBS. After treatment with 5% β-mercaptoethanol in SPM (1.2 M sorbitol; 50 mM K-phosphate, pH 7.3; and 1 mM MgCl_2_) for 1 h at 37°C with gentle agitation, cells were washed with SPM and then digested with 10 µl of zymolyase^TM^ (Zymo Research, 4 U/µL), 100 mg of lysing enzymes from *Trichoderma harzianum* (Sigma), and 0.1% bovine serum albumin (Sigma) in 1 ml of spheroplasting buffer (1 M sorbitol; 10 mM EDTA; and 100 mM sodium citrate, pH 5.8) for 40 min at 37°C with gentle agitation. Spheroplasts were gently harvested at 1600 rpm for 5 min at 4°C and washed three times with SPM to remove the enzymes. Samples were permeabilized with 0.5 ml of fresh prepared 0.1% Triton X-100, 0.1% sodium citrate solution in cold SPM for 2 min in ice, washed twice with SPM, and incubated with 50 µl TUNEL reaction mixture (In Situ Cell Death Detection Kit, Roche) for 60 min at 37°C in dark and humid conditions. Cells were washed with PBS and immediately analyzed by epifluorescence microscopy (see next section) or flow cytometry. TUNEL positive cells were quantified by collecting 10,000 events on the FL1 (FITC) channel of a FACSCalibur flow cytometer (Becton Dickinson) using CellQuest software (Becton Dickinson). Cell debris were excluded using the side and forward scatter dot-plot and a negative control (fixed and permeabilized wild-type cells incubated in 50 µl of TUNEL solution without terminal transferase) and a positive control [fixed and permeabilized wild-type cells treated with 0.1 µg/µl DNase I (Sigma) for 10 min at room temperature to induce DNA strand breaks prior to TUNEL labeling] were used to determine the percentage of TUNEL positive cells.

### Annexin V assay

Exposed phosphatidylserine was detected by reaction with FITC-coupled Annexin V (Apoptosis Detection kit, Oncogene Research Products). Log-phase cultures diluted to an OD_600_ of 0.1 were treated with 2 mM H_2_O_2_ (Sigma) for 3 h at 37°C. Yeast cells were washed with PBS, treated with 5% β-mercaptoethanol, and spheroplasted as described above for the TUNEL assay. Spheroplasts were washed two times with SPM and once with Binding Buffer diluted to 1x with 2 M sorbitol, and double stained with FITC-Annexin V and propidium iodine (PI) for 20 min at room temperature in the dark dark as described by [77]. Annexin V-positive, PI-negative cells were quantified by collecting 10,000 events on the FL1 (FITC) and FL2 (PE) channels of a FACSCalibur flow cytometer (Becton Dickinson) using CellQuest software (Becton Dickinson).

### Microscopy

To evaluate TUNEL assays, cells were mounted in ProLong Gold antifade reagent with DAPI (Molecular Probes). Brightfield, differential interference microscopy (DIC), and fluorescence images were captured with a Zeiss Axio Imager widefield fluorescence microscope (Carl Zeiss) equipped with an Orca cooled-CCD camera (Hamamatsu) using DAPI and FITC channels. Images were interfaced with MetaMorph software (Universal Imaging) and processed with PhotoShop (Adobe).

Mating structures were captured with an Eclipse E400 microscope (Nikon) equipped with a DXM1200F digital camera (Nikon). Images were interfaced with ACT-1 software (Nikon) and processed with PhotoShop (Adobe). For scanning electron microscopic analysis, mating plates were processed at the North Carolina State University Center for Electron Microscopy, Raleigh, NC, USA. Areas of interest were excised intact from the agar and fixed with 0.1 M sodium cacodylate (pH 6.8) buffer containing 3% glutaraldehyde for several days at 4°C. Samples were then washed for 1 h in cold sodium cacodylate buffer three times, dehydrated through a graded series of cold 30% and 50% ethanol for 1 h each, and held overnight in 70% ethanol. Dehydration was completed with 1-h incubations with cold 95% and 100% ethanol at 4°C, warming to room temperature in the 100% ethanol, followed by two additional 1-h washes with 100% ethanol at room temperature. The dehydrated samples were critical-point dried with liquid CO_2_ in a SAMDRI-795 (Tousimis) for 15 min and mounted on stubs with silver paint to ensure good adhesion and conductivity. Finally, the samples were coated with 50 Å of gold/palladium using a Hummer 6.2 sputter coater (Anatech) and held in a vacuum desiccator until viewed at 15 kV on a JSM 5900LV scanning electron microscope (JEOL), photographed with a Digital Scan Generator (JEOL) image acquisition system, and processed with PhotoShop (Adobe).

### Etest

In vitro antifungal susceptibility to FLC and AMB was determined by Etest (AB Biodisk) according to the manufacturer's instructions. Cells grown to mid-log phase were washed in 0.85% NaCl and diluted to an OD_600_ of 0.5 (∼1 McFarland turbidity). Inocula were applied with cotton swabs in RPMI-1640 agar (Sigma) supplemented with 2% glucose and buffered to pH 7.0 with MOPS and allowed to dry completely before applying the Etest strip. Plates were incubated at 35°C for 72 h and the MIC was determined as the first growth inhibition ellipse.

### Isolation of fluconazole resistant strains

Distinct colonies within the inhibition halo around the FLC strip during Etest assays were replica plated onto YPD agar and YPD agar plus 32 µg/ml FLC and incubated at 30°C. Isolates able to grow on the FLC plate after 2 days were inoculated into 5 ml YPD liquid and grown overnight at 30°C with agitation. Liquid cultures were tested by Etest assays and used to make gDNA (later used for ploidy analysis) and −80°C glycerol stocks. For virulence experiments and FLC resistance stability assays, −80°C glycerol stocks were inoculated into 5 ml YPD liquid and grown overnight at 30°C with agitation.

### Array comparative genomic hybridization

Genomic DNA was extracted using alkyltrimethyl ammonium bromide buffer (CTAB) as described by [78]. The quality of the purified DNA was examined on an agarose gel and quantified in a Qubit fluorometer (Invitrogen) using the Quant-iT dsDNA BR kit according to Invitrogen's instructions. Then 2.5 µg of DNA was sonicated to generate ∼500-bp fragments, which were labeled with Perkin Elmer Cy3-dUTP (reference DNA from H99 wild-type strain) or Cy5-dUTP (strain of interest) using the Random Primer Reaction of BioPrime Array CGH Genomic Labeling System (Invitrogen). Labeled reference and sample DNA were combined in a 1∶1 ratio, purified using Microcon 30K Centrifugal Filter Units (Millipore), and concentrated in a Speed Vac Plus SC110 (Savant Instruments). Samples were resuspended in 50 µl of Long Oligo Hybridization Solution (Corning), denatured at 95°C for 5 min, and applied to the *C. neoformans* whole genome 70-mer oligonucleotide spotted array version II (Washington University Genome Sequencing Center) covered with a LifterSlip Microarray Coverslip (Erie Scientific Company). Arrays were hybridized at 42°C for 16 h and subsequently washed twice with 1X SSC, 0.3% SDS heated to 42°C, followed by two washes in 0.2X SSC, and two final washes with 0.05X SSC at room temperature for two minutes each. Slides were dried by centrifugation, scanned with a GenePix 4000B scanner (Axon Instruments), and analyzed using GenePix Pro 6.0 software (Molecular Devices). GeneSpring GX 7.3 (Agilent Technologies) was used to perform Lowess normalization of the data, and an S-plus script was used to remove exon-junction probes and to log2 transform the red/green ratios. The transformed data from experimental arrays and from four control hybridizations (Cy3-reference strain DNA x Cy5-reference strain DNA) were analyzed with CGH-Miner software [79], which uses the Clusters Along Chromosomes method to identify DNA copy number alterations. Raw data for the regions of gain/loss identified by the algorithm were then manually inspected to confirm the calls or eliminate occasional errors induced by noise. The plots of log2 data as a function of chromosomal nucleotide position were created using a sliding-window of 10 consecutive probes and a targeted false discovery rate (FDR, i.e., the expected proportion of false positive results) of 0.01. Vertical bars were plotted in red for amplifications and green for deletions that are statistically significant. Vertical bars in gray indicate no DNA copy number alteration or amplifications or deletions that did not meet the statistical settings.

### Fluconazole resistance and reversion rates

FLC resistance rates were calculated by fluctuation analysis using the method of the median [80]. To measure FLC resistance rates, individual colonies of each strain were grown non-selectively in YPD agar from −80°C glycerol stocks. Twelve independent cultures from isolated colonies were grown in YPD liquid medium overnight at 30°C. Yeast cells were collected by centrifugation, washed with water, and serial dilutions were plated on YPD agar and YPD agar with 32 µg/ml FLC to determine the number of viable cells and the number of FLC^R^ cells, respectively. The median number of FLC^R^ cells in the different cultures was corrected for dilution factors, fractions plated, and number of viable cells. The numbers of FLC^R^ cells were ranked, and the 3^rd^ and 10^th^ ranks were used to calculate the lower and upper limits of the 95% confidence interval [81]. Strains resistant to FLC were used to calculate the reversion rates after approximately 20 generations. Non-selectively grown cultures from -80°C glycerol stocks were incubated at 30°C for 24 h in YPD liquid. Culture dilutions were plated on YPD agar, and 100 individual colonies were replica plated onto YPD agar and YPD agar plus 32 µg/ml FLC, followed by incubation at 30°C. Colonies unable to grow on the FLC plate after 2 days were considered revertants.

### Virulence studies

Virulence assays were conducted using a murine inhalation model of cryptococcosis. Cohorts of 4- to 6-week-old female A/JCr mice were anesthetized by intraperitoneal injection of Nembutal (37.5 mg/kg) and infected intranasally with 5×10^4^ cells in 50 µl of PBS pipetted slowly into the nares. Inoculum concentration was confirmed by plating serial dilutions in YPD agar and counting colony forming units (CFU) after 2 days. Mice were monitored twice daily, and those that showed signs of severe morbidity (weight loss, extension of the cerebral portion of the cranium, abnormal gait, paralysis, seizures, convulsions, or coma) were sacrificed by CO_2_ inhalation. The survival rates of animals were plotted against time, and p-values were calculated with the Mann-Whitney U test. In the first experiment using FLC treatment (Figure S5), 10 mice per strain were treated with 20 mg/kg/day FLC intraperitoneally, while control groups of 10 mice received only saline. Treatment started 24 h after inoculation of yeasts and was continued for 14 days. In the second experiment using FLC treatment (Figure 3C), 10 mice of about 20 g each were treated with 100 mg/kg/day FLC in the sole source of drinking water as described by [35]. Water intake was recorded daily for each cage (all 5 mice were assumed to drink the same volume) and averaged 0.23 ml/g. Treatment started 24 h after infection and was continued through day 60 with water being replaced every 2 to 3 days. FLC concentrations for each cage were recalculated based on weights of the animals and the water intake measured during the preceding days. Three euthanized mice in each treated group were dissected on the days indicated post-infection; their brains and lungs were removed, weighed, and homogenized in 1 ml sterile PBS. Serial dilutions of the organ samples were plated on Sabouraud-dextrose agar plus 100 µg/ml chloramphenicol and incubated at 30°C. Up to 50 individual colonies were replica plated onto YPD agar and YPD agar plus 32 µg/ml FLC and incubated at 30°C. Colonies unable to grow on the FLC plate after 2 days were considered revertants. Isolates able to grow on FLC were further tested by aCGH, qPCR, and Etest assays as described above.

### RNA extraction and cDNA synthesis

Yeast cells for RNA extraction were grown in YPD liquid culture overnight and were harvested the following day and washed. RNA preparations were isolated with the RNAeasy Mini Kit (Qiagen), including the DNase treatment step, and reverse transcriptase reactions were performed using AffinityScript RT-RNase (Stratagene).

### Quantitative PCR and RT-PCR analysis

Quantitative PCR reactions were performed in an Applied Biosystems 7500 Real-Time PCR System using Brilliant SYBR Green qRT-PCR master mix (Stratagene). PCR thermal cycling conditions were an initial step at 95°C for 5 min followed by 40 cycles at 95°C for 15 s, 60°C for 20 s and 72°C for 20 s. In each assay, "no-template" controls were included and melting curve analysis was performed to confirm a single PCR product. All experiments were done in triplicate for both the gene of interest and the control gene (*GPB1* or *ACT1*, see figure legends). Data were normalized to the control genes and relative expression was determined by the 2^−ΔΔCT^ method. The sequences of primers used are listed in Table S2. Detection of Chr1 disomy by qPCR was adapted from the method described by [81].

### Mating cell fusion assays

To perform the mating cell fusion assays, wild-type and mutant strains containing HYG, NAT, or NEO resistance genes were grown in YPD liquid overnight. Yeast cells were washed and adjusted to 5×10^6^ cells/ml, mixed 1∶1, and grown on V8 medium (pH 5.0) in the dark for 48 h. The mating colonies were collected by scraping, resuspended in sterile water, and serial dilutions were plated onto YPD medium containing hygromycin, nourseothricin, and/or G418 to select for cell-cell fusion products that have two drug-resistance markers (one from either parent). Plates were incubated at 30°C for 3 days until colonies formed and were then counted. The average number of colonies in triplicate assays was calculated and the fusion efficiency in apoptotic-mutants was determined as a function of the wild-type.

### Competitive fitness and growth curve assays

For competition assays, overnight cultures in YPD were washed three times with sterile water and adjusted to a density of 1×10^7^ cells/ml. The following strains were paired in a 1∶1 ratio: H99 and CPS76 (H99 HYG); H99 and CPS3; H99 and YPH104; and H99 and CPS89. Each mixture was inoculated onto fresh YPD medium and incubated at 30°C for the indicated times. Fifty isolated colonies from each time point were replica plated onto selective media and survival rates were calculated. Growth curves were determined by diluting the strains to OD_600_ of 0.01 in YPD and incubation at 30°C without agitation. The turbidity of quadruplicate assays was measured at OD_600_ every hour with a microdilution plate reader (Sunrise, Tecan). Readings were corrected for background (YPD media, no cells added), averaged, and plotted versus time in Excel (Microsoft Office).

### Statistical analysis

All quantitative data are reported as means ± standard deviation and are derived from at least three independent experiments. The significance of the data was assessed using two-tailed t-tests to compare mutants with the wild-type strain. For comparisons of mice survival data, the logrank test was performed using Prism 4 (GraphPad Software). A p-value of less than 0.05 was considered significant.

### Accession numbers

*AIF1* (CNAG_04521); *BUB1* (CNAG_03184); *COX1* (CNAG_09009); *ENDOG* (CNAG_02204); *IAP1* (CNAG_04708); *MCA1* (CNAG_04636), *MCA2* (CNAG_06787), and *AIF1* from RCT 17 strain (JN606083).

## Supporting Information

Figure S1 ***C. neoformans*** **cells treated with hydrogen peroxide display apoptotic phenotypes, but apoptotic markers can not be detected after treatment with amphotericin B and fluconazole. A.** Annexin V assay indicating externalization of phosphatidylserine (PS) after treatment with 2 mM H_2_O_2_ . Wild-type H99 strain and the *aif1*, *mca1 mca2*, and *aif1 mca1 mca2* mutant strains were treated with 2 mM H_2_O_2_ for 3 hr, double stained with FITC-labeled Annexin V and propidium iodide (PI) and analyzed by flow cytometry. Percentages of Annexin V-positive/PI-negative cells (early apoptotic cells), Annexin V-negative/PI- positive cells (necrotic cells), and Annexin V/PI-double-positive cells (late apoptotic or necrotic cells) are indicated. Data represent mean ± standard deviation. ** and *** indicates significance at p<0.01 and p<0.001 compared to WT. **B.** Representative flow cytometry pictograms of cells that were treated with 2 mM H_2_O_2_, 0.125 µg/ml AMB or 64 µg/ml FLC and labeled with TUNEL assay (the percentage of TUNEL-positive cells is indicated inside each panel).
(EPS)

Figure S2 **Apoptotic mutants are more resistant to H_2_O_2_.** Two independent *aif1*, *mca1 mca2*, and *aif1 mca1 mca2* mutants were grown on YPD media with 0 or 2 mM H_2_O_2_ and incubated at 37°C for 2 days.
(EPS)

Figure S3 **qPCR analysis of FLC^R^ strains described in** **Table 3** **. A.** Position of *ERG11*, *ACT1*, and *AFR1* genes on Chr1. **B.** Copy number of the indicated genes on Chr1 determined by qPCR. Relative gene copy number represent mean ΔCt values normalized to *GPB1* (CNAG_06699; chromosome 7) and relative to copy number values in the H99 wild-type strain (value arbitrarily set to 1). Bars represent standard deviation for triplicates.
(EPS)

Figure S4 **aCGH analysis of FLC^R^ strains described in** **Table 3** **.** The log2 of the ratio of the hybridization signal of genomic DNA from the indicated FLC^R^ strains relative to the hybridization signal from H99 wild-type strain were plotted as a function of its chromosomal location. Each chromosome was color-coded and their numbers are listed at the bottom.
(EPS)

Figure S5 **Virulence assay and efficacy of fluconazole therapy in murine model of cryptococcosis.** FLC^R^ clones are more virulent than wild-type and *aif1* mutant backgrounds in the presence of FLC treatment of murine cryptococcal infections. Mice were infected intranasally with strains H99, CPS3, CPS18, and CPS24 and treated with 20 mg/kg/day of FLC.
(EPS)

Figure S6 **Fluconazole sensitivity of different strains using Etest assays. A.** Reconstitution of Aif1 complements the increased fluconazole heteroresistance of RCT 17 strain. **B.** Etest strip assays showing that an *aif1* diploid strain (CPS80) exhibits a higher frequency of the intrinsic adaptive fluconazole heteroresistant phenotype when compared to a wild-type diploid strain (CPS83).
(EPS)

Figure S7 **Apoptotic-defective mutants show reduced mating and defective secretion of mating pheromone. A.** Mating assays between the indicated α and **a** strains on V8 medium (pH 5.0, upper panels) and MS agar (lower panels) incubated for 14 days at room temperature in the dark, showing that mutants produce less hyphae than the wild-type strain during bilateral crosses. Plates were observed at 40X magnification to visualize mating hyphae and basidia. **B.** Apoptotic defective strains were confronted with opposite mating type *crg1* mutant strains on V8 medium (pH 5.0) and incubated for 7 days at room temperature in the dark. The apoptotic mutant strains stimulated less conjugation tube formation by the *crg1* strains.
(EPS)

Figure S8 **Apoptotic-defective mutants exhibit cell fusion defects during mating.** Fusion products from the indicated crosses were selected on YPD medium containing hygromycin, nourseothricin, and/or G418. Plates were observed at 40X magnification to visualize the formation of hyphae by the fusion products. Fusion efficiency was determined by counting the number of visible large colonies. Strains from the left: YSB119 x YSB121; CPS70 x CPS15; YPH90 x CPS11; CPS9 x CPS5; and CPS76 x CPS13.
(EPS)

Figure S9 **Loss of apoptosis-like cell death does not influence growth rate.** Growth curves of the indicated strains in liquid YPD medium at 30°C without agitation. Bars represent standard deviation for quadruplicate assays.
(EPS)

Table S1 Strains used in this study.
(DOCX)

Table S2 Primers used in this study
(DOCX)
