# Supplementary material for: Deletion of Cryptococcus neoformans AIF Ortholog Promotes Chromosome Aneuploidy and Fluconazole-Resistance in a Metacaspase-Independent Manner
Source: PLoS Pathog. 2011 Nov 17;7(11):e1002364. doi: 10.1371/journal.ppat.1002364 (PMC3219705; doi:10.1371/journal.ppat.1002364)
Supplement: Table S1 — Strains used in this study. (DOCX) [file ppat.1002364.s010.docx]

**Table S1.** Strains used in this study.

| **Strain name** | **Genotype** | **Background** | **Reference** |
| --- | --- | --- | --- |
| H99 | Aα | Wild-type | [1] |
| KN99**a** | A**a** | Wild-type | [2] |
| YL99**a** | A**a** | H99 x KN99**a** | This study |
| CPS3 | Aα *aif1::NAT* | H99 | This study |
| CPS5 | A**a** *mca1::NAT* | YPH92 x KN99**a** | This study |
| CPS9 | Aα *mca2::NEO* | YPH104 x KN99**a** | This study |
| CPS11 | A**a** *mca2::NEO* | YPH104 x KN99**a** | This study |
| CPS13 | A**a** *mca1::NAT mca2::NEO* | YPH104 x KN99**a** | This study |
| CPS15 | A**a** *aif1::NAT* | CPS3 x KN99**a** | This study |
| CPS18 | Aα *aif1::NAT* FLC^R256^ n + (1) | CPS3 | This study |
| CPS21 | Aα *aif1::NAT* FLC^R256^ n + (1) | CPS3 | This study |
| CPS24 | Aα *aif1::NAT* FLC^R256^ n + dup(1)(3) | CPS3 | This study |
| CPS26 | Aα *aif1::NAT* FLC^R256^ n + (1) | CPS3 | This study |
| CPS28 | Aα *aif1::NAT* FLC^R256^ n + (1) | CPS3 | This study |
| CPS35 | A**a** *aif1::NAT* FLC^R256^ n + (1) | CPS15 | This study |
| CPS37 | A**a** *aif1::NAT* FLC^R256^ n + (1) | CPS15 | This study |
| CPS48 | Aα *aif1::NAT* FLC^R256^ n + (1)(4) | CPS3 | This study |
| CPS49 | Aα *aif1::NAT* FLC^R256^ n + (1) | CPS3 | This study |
| CPS51 | Aα FLC^R32^ | H99 | This study |
| CPS52 | Aα FLC^R16^ | H99 | This study |
| CPS53 | Aα FLC^R64^ n + (1) | H99 | This study |
| CPS70 | Aα *aif1::HYG* | H99 | This study |
| CPS76 | Aα HYG | H99 | This study |
| CPS80* | A *aif1::NAT aif1::HYG* 2n | CPS70 x CPS13 | This study |
| CPS83* | A 2n | YSB119 x YSB121 | This study |
| CPS89 | Aα *aif1::HYG mca1::NAT mca2::NEO* | CPS70 x CPS13 | This study |
| CPS104 | Aα FLC^R256^ n + (1) | RCT 17 | This study |
| CPS105 | Aα FLC^R32^ | H99 | This study |
| CPS106 | Aα FLC^R256^ n + (1) | CPS51 | This study |
| CPS107 | Aα FLC^R256^ n + (1) | CPS51 | This study |
| CPS108 | Aα FLC^R192^ n + (1) | CPS52 | This study |
| CPS109 | Aα FLC^R192^ n + (1) | CPS52 | This study |
| CPS110 | Aα *aif1::HYG* FLC^R256^ n + (1) | CPS70 | This study |
| CPS111 | Aα *aif1::HYG* FLC^R256^ n + (1) | CPS70 | This study |
| CPS114 | Aα *aif1::HYG* FLC^R256^ n + (1) | CPS70 | This study |
| CPS116 | A**a** *aif1::HYG mca1::NAT mca2::NEO* | CPS70 x CPS13 | This study |
| CPS126 | Aα *aif1::HYG* FLC^R256^ n + (1) | CPS70 | This study |
| CPS128 | Aα *aif1::HYG* FLC^R256^ n + (1) | CPS70 | This study |
| CPS129** | Aα FLC^R96^ 2n + (1) | H99 | This study |
| CPS134** | Aα *aif1::NAT* FLC^R128^ n + (1)(4)(6) | CPS3 | This study |
| CPS135 | Aα *aif1::HYG* FLC^R256^ n + (1) | CPS70 | This study |
| CPS136 | Aα FLC^R64^ n + (1) | RCT 17 | This study |
| CPS137 | Aα FLC^R24^ | H99 | This study |
| CPS141 | Aα FLC^R256^ n + (1) | RCT 17 | This study |
| CPS145** | Aα *aif1::NAT* FLC^R256^ n + (1) | CPS18 | This study |
| CPS146** | Aα *aif1::NAT* FLC^R256^ n + dup(1)(3) | CPS24 | This study |
| CPS147 | Aα FLC^R256^ n + (1) | CPS105 | This study |
| CPS148 | Aα FLC^R256^ n + (1) | CPS108 | This study |
| CPS149 | Aα FLC^R256^ n + (1) | CPS109 | This study |
| CPS151 | A**a** FLC^R48^ n + (1) | KN99**a** | This study |
| CPS152 | Aα FLC^R256^ n + (1) | RCT 17 | This study |
| CPS153 | Aα FLC^R256^ n + (1) | RCT 17 | This study |
| CPS154 | A**a** FLC^R48^ n + (1) | YL99**a** | This study |
| CPS155 | A**a** FLC^R64^ n + (1) | YL99**a** | This study |
| CPS157 | Aα FLC^R256^ n + (1) | RCT 17 | This study |
| CPS158 | Aα FLC^R256^ n + (1) | RCT 17 | This study |
| CPS159 | Aα FLC^R256^ n + (1) | RCT 17 | This study |
| CPS160 | Aα FLC^R192^ n + (1) | RCT 17 | This study |
| CPS161 | A**a** *bub1::NAT* | YL99**a** | This study |
| CPS174 | Aα *aif1::NAT AIF1-NEO* | CPS3 | This study |
| CPS175 | Aα *AIF1-NEO* | RCT 17 | This study |
| CPS177 | Aα *aif1::NAT AIF1-NEO* FLC^R16^ | CPS174 | This study |
| CPS178 | Aα *aif1::NAT AIF1-NEO* FLC^R48^ n + (1) | CPS174 | This study |
| CPS179 | Aα *aif1::NAT AIF1-NEO* FLC^R48^ n + (1) | CPS174 | This study |
| CPS180 | Aα *AIF1-NEO* FLC^R32^ | CPS175 | This study |
| CPS181 | Aα *AIF1-NEO* FLC^R96^ n + (1) | CPS175 | This study |
| CPS182 | Aα *AIF1-NEO* FLC^R64^ n + (1) | CPS175 | This study |
| CPS183 | Aα *aif1::NAT AIF1-NEO* FLC^R8^ | CPS174 | This study |
| CPS184 | Aα *aif1::NAT AIF1-NEO* FLC^R8^ | CPS174 | This study |
| CPS185 | Aα *aif1::NAT AIF1-NEO* FLC^R24^ | CPS174 | This study |
| CPS186 | Aα *AIF1-NEO* FLC^R32^ | CPS175 | This study |
| CPS187 | Aα *AIF1-NEO* FLC^R16^ | CPS175 | This study |
| CPS188 | Aα *AIF1-NEO* FLC^R32^ | CPS175 | This study |
| CPS189 | Aα *aif1::NAT AIF1-NEO* FLC^R16^ | CPS174 | This study |
| CPS190 | Aα *aif1::NAT AIF1-NEO* FLC^R8^ | CPS174 | This study |
| CPS191 | Aα *AIF1-NEO* FLC^R16^ | CPS175 | This study |
| CPS192 | Aα *AIF1-NEO* FLC^R32^ | CPS175 | This study |
| CPS193 | Aα *aif1::NAT AIF1* FLC^R16^ | CPS174 | This study |
| CPS194 | Aα *aif1::NAT AIF1* FLC^R32^ | CPS174 | This study |
| CPS195 | Aα *AIF1-NEO* FLC^R32^ | CPS175 | This study |
| CPS196 | Aα *AIF1-NEO* FLC^R32^ | CPS175 | This study |
| HC-4*** | Aα | Clinical isolate | This study |
| HC-6*** | Aα | Clinical isolate | This study |
| HC-9*** | Aα | Clinical isolate | This study |
| KK8 | A**a** *cna1::NEO* | KN99**a** | [3] |
| RCT 17*** | Aα | Clinical isolate | This study |
| RCT 50*** | Aα | Clinical isolate | This study |
| RCT 52*** | Aα | Clinical isolate | This study |
| RCT 55*** | Aα | Clinical isolate | This study |
| YPH92 | Aα *mca1::NAT* | H99 | This study |
| YPH104 | Aα *mca1::NAT mca2::NEO* | H99 | This study |
| YPH276 | Aα *crg1::NAT* | H99 | [4] |
| YPH570 | A**a** *crg1::NAT* | KN99**a** | [4] |
| YSB119 | Aα *aca1::NAT* *ura5 ACA1-URA5* | H99 | [5] |
| YSB121 | A**a** *aca1::NEO* *ura5 ACA1-URA5* | KN99**a** | [5] |

*Diploid strains are fusion products of the indicated strains selected for double drug resistance. Their ploidy was confirmed by flow cytometry experiments.

**FLC^R^ colonies isolated from lungs (CPS129 and CPS134) and brains (CPS145 and CPS146) of mice inoculated with the indicated background strains and treated with 100 mg/kg/day of FLC.

***Clinical isolates from the cerebrospinal fluid (CSF) of patients with cryptococcal meningitis from USA (HC isolates, Duke patients) and from South Africa (RCT isolates, obtained from Tihana Bicanic and Tom Harrison).

**References**

1. Perfect JR, Ketabchi N, Cox GM, Ingram CW, Beiser CL (1993) Karyotyping of *Cryptococcus neoformans* as an epidemiological tool. J Clin Microbiol 31: 3305-3309.

2. Nielsen K, Cox GM, Wang P, Toffaletti DL, Perfect JR, et al. (2003) Sexual cycle of *Cryptococcus neoformans* var. *grubii* and virulence of congenic a and alpha isolates. Infect Immun 71: 4831-4841.

3. Kojima K, Bahn YS, Heitman J (2006) Calcineurin, Mpk1 and Hog1 MAPK pathways independently control fludioxonil antifungal sensitivity in *Cryptococcus neoformans*. Microbiology 152: 591-604.

4. Hsueh YP, Xue C, Heitman J (2009) A constitutively active GPCR governs morphogenic transitions in *Cryptococcus neoformans*. EMBO J 28: 1220-1233.

5. Bahn YS, Hicks JK, Giles SS, Cox GM, Heitman J (2004) Adenylyl cyclase-associated protein Aca1 regulates virulence and differentiation of *Cryptococcus neoformans* via the cyclic AMP-protein kinase A cascade. Eukaryot Cell 3: 1476-1491.
