# Supplementary material for: Deletion of Cryptococcus neoformans AIF Ortholog Promotes Chromosome Aneuploidy and Fluconazole-Resistance in a Metacaspase-Independent Manner
Source: PLoS Pathog. 2011 Nov 17;7(11):e1002364. doi: 10.1371/journal.ppat.1002364 (PMC3219705; doi:10.1371/journal.ppat.1002364)
Supplement: Table S2 — Primers used in this study (DOCX) [file ppat.1002364.s011.docx]

**Table S2.** Primers used in this study

| **Primer** | **Sequence (5' to 3')** | **Description** |
| --- | --- | --- |
| JOHE21113 | GGATGGCATTACATCTTGCTC | H99 *AIF1* disruption |
| JOHE21255 | CTGGCCGTCGTTTTACAACTGTATGCTCAAAATGAGGTTG | H99 *AIF1* disruption |
| JOHE21258 | GTCATAGCTGTTTCCTGGCAGTAATGTTAAAATCCCTCCA | H99 *AIF1* disruption |
| JOHE21259 | CGAAGAAAGCTACGCGCTAC | H99 *AIF1* disruption |
| JOHE21181 | CGGAGTTTTTTCTCGTGCTAACG | *aif1* allele screening |
| JOHE21182 | CTTGACACTCACTCATCTCGGAATC | *aif1* allele screening |
| JOHE22748 | TCTAGAGGATGGCATTACATCTTGCTC | H99 *AIF1* complementation |
| JOHE22749 | CTCGAGCGAAGAAAGCTACGCGCTAC | H99 *AIF1* complementation |
| JOHE15510 | AGACCCTGGCTGACAGTCCC | H99 *MCA1* disruption |
| JOHE15511 | CTGGCCGTCGTTTTACAGCTGAATGTTTATAGCTGGT | H99 *MCA1* disruption |
| JOHE15512 | GTCATAGCTGTTTCCTGGAAGCAAATTGCCATATATT | H99 *MCA1* disruption |
| JOHE15513 | ACGGACCATGTAACTGTAGA | H99 *MCA1* disruption |
| JOHE15514 | GTGGATGATGACAGTGAGTG | *mca1* allele screening |
| JOHE15515 | GAACAGGATGTTGGTATCTA | *mca1* allele screening |
| JOHE16178 | CGACACCCTTAATCGTCTGA | H99 *MCA2* disruption |
| JOHE16179 | CTGGCCGTCGTTTTACATTCTACTGCCCATCAAATTGC | H99 *MCA2* disruption |
| JOHE16180 | GTCATAGCTGTTTCCTGGTAGTAGGAGGTTGGTGCAT | H99 *MCA2* disruption |
| JOHE16181 | CTTCCACCTCGGCCTCCGCC | H99 *MCA2* disruption |
| JOHE16182 | GGAAGGCCTCACCCTCAGCA | *mca2* allele screening |
| JOHE16183 | ATCAGGCTCTTTGACAGTGC | *mca2* allele screening |
| JOHE26472 | AACGTACACATTGGGATCTTCA | H99 *BUB1* disruption |
| JOHE26473 | CTGGCCGTCGTTTTACCTTCGAATTGTCGGAAGACAC | H99 *BUB1* disruption |
| JOHE26474 | GTCATAGCTGTTTCCTGAGTCCGCGTTAGAGGAGGAG | H99 *BUB1* disruption |
| JOHE26475 | AACTCTGCCGGTATGTCCAC | H99 *BUB1* disruption |
| JOHE26476 | CCACAGGCGGGTAAACGCGA | *bub1* allele screening |
| JOHE26477 | ACCATTGCGAGTTTGGTAGG | *bub1* allele screening |
| JOHE24035 | AACGAATGTTCCTGCTACCC | H99 *AFR1* qPCR Fw |
| JOHE24036 | CCTCACGCTCTCTGGTCATA | H99 *AFR1* qPCR Rv |
| JOHE24037 | TGGTCGTCAAGGTTGGAATCA | H99 *GPD1* qPCR Fw |
| JOHE24038 | GTCGTTGACAGCAACAACCT | H99 *GPD1* qPCR Rv |
| JOHE24041 | TGCTTTCCCTCGATTTGC | H99 *AIF1* qPCR Fw |
| JOHE24042 | TTAGGGATGGCTGGTTCATT | H99 *AIF1* qPCR Rv |
| JOHE26368 | AAGCTTGCTGTTCCCAAGTT | H99 *ERG11* qPCR Fw |
| JOHE26369 | CCTATTTGACCTCGGCATTT | H99 *ERG11* qPCR Rv |
| JOHE19493 | ATCGTTCTTGACTCTGGTGACGGT | H99 *ACT1* qPCR Fw |
| JOHE19494 | AAGTGGTGAAGAGGTAACCACGCT | H99 *ACT1* qPCR Rv |
